# Higher Life-Course Blood Pressure Associates With Reduced Myocardial Perfusion in Older Age: Insights From MyoFit46

**DOI:** 10.1161/CIRCIMAGING.125.019105

**Published:** 2025-12-17

**Authors:** Constantin-Cristian Topriceanu, Matthew Webber, Hunain Shiwani, Fiona Chan, Emma Martin, Debbie Falconer, Matthew A. Stanley, Jonathan Bennett, Pablo Gonzalez-Martin, Haytham Shah, Swapnanil De, Andrew Wong, Iain Pierce, Rhodri H. Davies, Pier D. Lambiase, Nishi Chaturvedi, Peter Kellman, Rebecca Hardy, James C. Moon, Alun D. Hughes, Gabriella Captur

**Affiliations:** 1 Unit for Lifelong Health and Ageing at UCL (C.-C.T., M.W., H. Shiwani, F.C., E.M., D.F., M.A.S., J.B., H. Shah, S.D., A.W., R.H.D., N.C., A.D.H., G.C.), University College London, United Kingdom.; 2 UCL Institute of Cardiovascular Science (C.-C.T., M.W., H. Shiwani, F.C., E.M., D.F., M.A.S., J.B., P.G.-M., H. Shah, S.D., A.W., R.H.D., P.D.L., N.C., J.C.M., A.D.H., G.C.), University College London, United Kingdom.; 3 Cardiology Department, The Royal Free Hospital, Centre for Inherited Heart Muscle Conditions, London, United Kingdom (C.-C.T., G.C.).; 4 Mayo Clinic, Rochester, MN (C.-C.T.).; 5 Cardiac MRI Unit, Barts Heart Centre, London, United Kingdom (H. Shiwani, I.P., P.D.L., J.C.M.).; 6 ELEM Biotech SL, Barcelona, Spain (P.G.-M.).; 7 National Heart, Lung, and Blood Institute, National Institute of Health, Bethesda, MD (P.K.).; 8 School of Sport, Exercise and Health Sciences, Loughborough University, United Kingdom (R.H.).

**Keywords:** blood pressure, fibrosis, heart failure, myocardial infarction, perfusion

## Abstract

**BACKGROUND:**

Elevated blood pressure (BP) is a major contributor to coronary artery disease. We explored the impact of life-course BP on later-life normalized stress myocardial blood flow (sMBF_N_) and myocardial perfusion reserve by cardiovascular magnetic resonance (CMR).

**METHODS:**

MyoFit46 prospectively recruited ≈500 National Survey of Health and Development 1946 birth cohort participants, aged ≈77 years, to undergo stress perfusion and late gadolinium enhancement CMR. Systolic (SBPs) and diastolic BPs were recorded at 36, 43, 53, 63, 69, and 77 years. For each participant, the annual rates of BP change (steepness of BP increase) and area under the BP trajectory curve (cumulative life-course BP burden) were derived using mixed-effects models. The associations between BP measures and CMR metrics were tested using generalized linear and additive models, adjusted for antihypertensive use, demographics, lifestyle choices, and comorbidities. Cross-sectional associations between CMR metrics and major adverse cardiovascular events (myocardial infarction, stroke, and heart failure) were also tested. Mediation analyses explored the mechanistic pathways linking life-course BPs, myocardial perfusion, and myocardial fibrosis.

**RESULTS:**

Among 459 included MyoFit46 participants, each 10 mm Hg higher SBP at 36 to 69 years was associated with 3% to 6% lower sMBF_N_ by CMR at 77 years. At 43 to 63 years, as SBPs rose from 120 to 140 mm Hg, sMBF_N_ was 18% to 24% lower. Having a sustained higher SBP by 10 mm Hg from 36 to 77 years was associated with 11% (95% CI, 8–14) lower sMBF_N_ at 77 years. Each 1 mm Hg/y steeper SBP rise during age intervals 36 to 43, 43 to 53, 53 to 63, and 63 to 69 years was associated with 2% to 6% lower sMBF_N_ at 77 years, associations not conditional on baseline or final BPs in each age interval. Associations may be clinically relevant as each 1% lower sMBF_N_ was associated with 3% higher odds of major adverse cardiovascular events. sMBF_N_ mediated ≈20% to ≈40% of the associations between life-course SBPs and late gadolinium enhancement at 77 years. Results were similar for diastolic BP, myocardial perfusion reserve, or sMBF (not normalized).

**CONCLUSIONS:**

Higher life-course BPs, steeper increases, and greater cumulative BP burden associate with lower myocardial perfusion by CMR at 77 years, which can be linked with higher odds of major adverse cardiovascular events and greater myocardial fibrosis burden. This underscores the importance of early life BP screening and guiding hyperetension treatment based on longitudinal BP trajectories (rather than relying solely on cross-sectional BPs).

**REGISTRATION:**

URL: https://www.clinicaltrials.gov; Unique identifier: NCT05455125.

**Clinical Perspective**

Blood pressures (BPs) were recorded at 36, 43, 53, 63, 69, and 77 years in participants from the 1946 NSHD study (National Survey of Health and Development), the world’s longest-running birth cohort with continuous follow-up. MyoFit46 prospectively recruited ≈500 NSHD participants, aged ≈77 years, for deep phenotyping cardiovascular magnetic resonance imaging. Using life-course epidemiological data, we show that higher life-course BPs (as early as 36 years), steeper increases (regardless of initial or final BPs), and more years spent at higher BPs were associated with worse normalized stress myocardial blood flow (sMBF_N_) and myocardial perfusion reserve. In turn, worse sMBF_N_ could be linked to greater myocardial fibrosis burden by late gadolinium enhancement and higher odds of a prevalent major adverse cardiovascular events composite, consisting of myocardial infarction, stroke, or heart failure. The steepest declines in sMBF_N_ were observed when midlife systolic BPs (43–63 years) rose from 120 to 140 mm Hg. This emphasizes the importance of early life BP screening, integrating life-course BP trajectories for a more personalized cardiovascular risk stratification (rather than relying solely on cross-sectional BP readings), rigorous midlife BP control, and considering lower BP treatment thresholds or targets.

**See Editorial by Kwapong et al**

Cardiovascular magnetic resonance (CMR) enables the noninvasive quantitative measurement of myocardial blood flow (MBF) and myocardial perfusion reserve (MPR). Abnormal CMR perfusion is a powerful predictor of major adverse cardiovascular events (MACE), such as cardiovascular death, myocardial infarction (MI), or heart failure (HF).^1^ High blood pressure (BP) accelerates atherosclerosis, culminating in ischemia and infarction, while simultaneously increasing left ventricular (LV) mass (LVM), which impairs subendocardial perfusion, even in the absence of obstructive cardiovascular artery disease.^2^ Over the past decades, systolic (SBP) and diastolic BP (DBP) thresholds for initiating antihypertensives have progressively decreased from 160/100^3^ to 130/80 mm Hg in high-risk patients, according to the latest American College of Cardiology/American Heart Association (AHA) and European Society of Cardiology (ESC) guidelines.^4^ Despite these evolving thresholds, evidence from large epidemiological studies^5^ suggests that some individuals who are still classified as normotensive may be at higher cardiovascular risk. A meta-analysis of >1 million individuals revealed increased mortality in those with BPs >115/75 mm Hg.^6^ This suggests that the current threshold-based approach may inadequately capture cardiovascular risk, especially since BP naturally fluctuates over time and individuals experience various patterns of BP change during their life.

A paradigm shift in how we approach BP may be needed, yet a fundamental knowledge gap persists: "How do BP trajectories across the life-course relate to myocardial perfusion in older age?" It is unclear whether trajectory-based metrics, such as the steepness of BP increase and cumulative BP burden, serve as better predictors of impaired perfusion, beyond static cross-sectional BPs. Moreover, whether specific subgroups of individuals with distinct BP trajectories are at higher cardiovascular risk is unknown. The temporal dynamics of BP-related cardiovascular risk also remain less understood. Specifically, the identification of age periods during which BP elevations or steep BP increases exert the greatest impact on later-life myocardial perfusion represents an important unresolved question. Finally, the mechanistic pathways linking life-course BPs, LVM, perfusion, and fibrosis warrant further investigation. Using unique longitudinal life-course BPs (recorded from 36 to 77 years) and quantitative CMR stress perfusion data acquired at 77 years from MyoFit46, we sought to answer these questions.

## Methods

### Study Population

The National Survey of Health and Development (NSHD) is a birth cohort comprised of 5,362 individuals born in one week in March 1946 in Britain.^7^ The NSHD flow chart from 1946 to 2020 is presented in Figure S1, highlighting the number of participants who died, were lost to follow-up, withdrew from the study, or moved abroad. MyoFit46 (NCT05455125) is the prospective longitudinal CMR substudy of NSHD, aiming to understand how early life, midlife, and older age risk factors influence cardiovascular health after 75 years, using data from ≈500 prospectively enrolled NSHD participants. Active NSHD participants were not eligible to participate in MyoFit46 if they had implantable cardiac electronic devices (except for implantable loop recorders), claustrophobia, renal failure, severe asthma, or high-grade atrioventricular blocks. Among those eligible for MyoFit46 inclusion, we prioritized individuals who participated in the majority of the NSHD data collection sweeps. The full process was described elsewhere.^8^

### Data Availability

NSHD data are available from https://nshd.mrc.ac.uk/. For MyoFit46 collaborations, please access http://www.myofit46.com/.

### Ethical Approval

A favorable opinion was provided by the London Queen Square Research Ethics Committee (REC: 19/LO/1774, IRAS: 254776). Study members provided written informed consent to participate.

### BPs Between 36 and 77 Years

Sitting SBPs and DBPs were recorded by a trained healthcare professional during home, and from age 63 years onwards, home or clinic visits, with an appropriately sized cuff, when participants were aged 36 years (in 1982), 43 (in 1989), 53 (in 1999), ≈63 (between 2006 and 2010), 69 (in 2015), and ≈77 years (between 2020 and 2024 as part of MyoFit46). The mean age (±1 SD) at the 2006 to 2010 visit was 63±1 years, and 77±1 years at the 2020 to 2024 visit. At each visit, participants also completed questionnaires, reporting antihypertensive use. A Hawksley random zero sphygmomanometer was used in 1982 and 1989. Validated oscillometer devices were used at subsequent visits. To enable comparisons, measurements from the random zero sphygmomanometer were converted into equivalent automated oscillometer values using validated conversion equations.^9^ Mean arterial pressure (MAP) was calculated as the 1:2 SBP:DBP weighted average. Pulse pressure was calculated as the difference between SBP and DBP. We also derived BPs corrected for antihypertensive use at each visit, by adding 10 mm Hg to SBP and 5 mm Hg to DBP observed values,^10–12^ in those taking such medications. However, we primarily used unadjusted BPs, as these represent the actual BPs individuals experience, and therefore provide the most clinically meaningful reflection of BP status.

### CMR Data Analysis

The CMR imaging protocol is presented in the Supplemental Methods. For postprocessing purposes, LV was divided into 16 AHA segments.^13^

#### Volumetric Analysis

Long- and short-axis cines were postprocessed using an artificial intelligence pipeline,^14^ to derive LV structure and function metrics such as LV volumes or LVM, which was indexed to contemporaneous body surface area to yield the LVM index.

#### Tissue Characterization

T_1_ and extracellular volume (ECV) maps were analyzed using cvi42. Manual ECV fraction (%) was calculated as ECV=(Δ [1/T_1_ myocardial]/Δ [1/T_1_ blood]×[1−hematocrit]) for each AHA segment. The mean global ECV (%) was calculated as the average ECV across the 16 AHA segments. Full details are presented in the Supplemental Methods.

#### First-Pass Quantitative Myocardial Perfusion

To evaluate the presence of inducible myocardial ischemia, quantitative rest and stress perfusion images were acquired using a dual-cannula approach.^15^ Inline automatic reconstruction and postprocessing of stress and rest first pass perfusion data were implemented with Gadgetron,^16^ excluding myocardial fat and papillary muscles. This allowed us to derive absolute stress (sMBF_1–16_) and rest MBFs (rMBF_1–16_), where each pixel encoded MBF in ml/g/min. Central BPs recorded at the time of CMR perfusion acquisitions were used to derive the rate pressure product (where rate pressure product=heart rate×central BP), allowing the normalization (_N_) of MBF (yielding rMBF_1-__16__N_ | sMBF_1-__16__N_). Central BP measurements were derived from the analysis of the brachial artery BP and supra-systolic pressure waveforms.^17^ Central BP was used in preference to peripheral brachial BP for myocardial blood flow normalization, since it has been previously shown to be a stronger independent predictor of cardiovascular outcomes.^18^

The global MBFs were calculated as the average of the corresponding 16 AHA segment values. Each LV segment was further subdivided into a 50% subendocardial and 50% subepicardial region, allowing the quantification of sMBFepi, sMBFepi_N_, sMBFendo, sMBFendo_N_, and equivalent rMBF metrics. The global LV and segment-specific MPR (MPR_1–16_) were calculated as sMBF/rMBF or sMBF_1-16_/rMBF_1-16_, respectively.

#### Quantification of Late Gadolinium Enhancement

In cvi42, the endocardial and epicardial borders on short-axis late gadolinium enhancement (LGE) images were manually drawn. LGE extent was measured semiautomatically by signal thresholding, using the 3SD method. Absolute LGE mass (expressed in grams) and LGE (%) were calculated per AHA segment, and averaged across all 16 segments to calculate global values.

### Mortality

All-cause mortality was recorded in NSHD participants, as they were flagged for death notification on the National Health Service Central Register.

### Covariates

Although this is a birth cohort and all participants were born during the same week in March 1946, age at CMR was calculated as the date of the CMR minus the date of birth. Sex was assigned at birth as male or female. At 36, 43, 53, and 63 years, participants’ socio-economic position was recorded according to the UK Office of Population Censuses and Surveys Registrar General’s social class, dichotomized as manual or nonmanual. At 36, 43, 53, 63, and 69 years, the body mass index was calculated as weight/height^2^, and participants completed questionnaires providing data on smoking status (recorded as current, ex-smoker, or never smoked), physical activity (age-specific options recorded in Table 1), and diabetes (yes/no). At 77 years, body mass index was calculated as weight/height^2^, and participants completed a questionnaire wherein they reported smoking status (yes/no) and their current medications and comorbidities (diabetes, MI, stroke, and HF, among others). These self-reported data were augmented using hospital or primary care records, submitted to NSHD across the life-course.

**Table 1.**
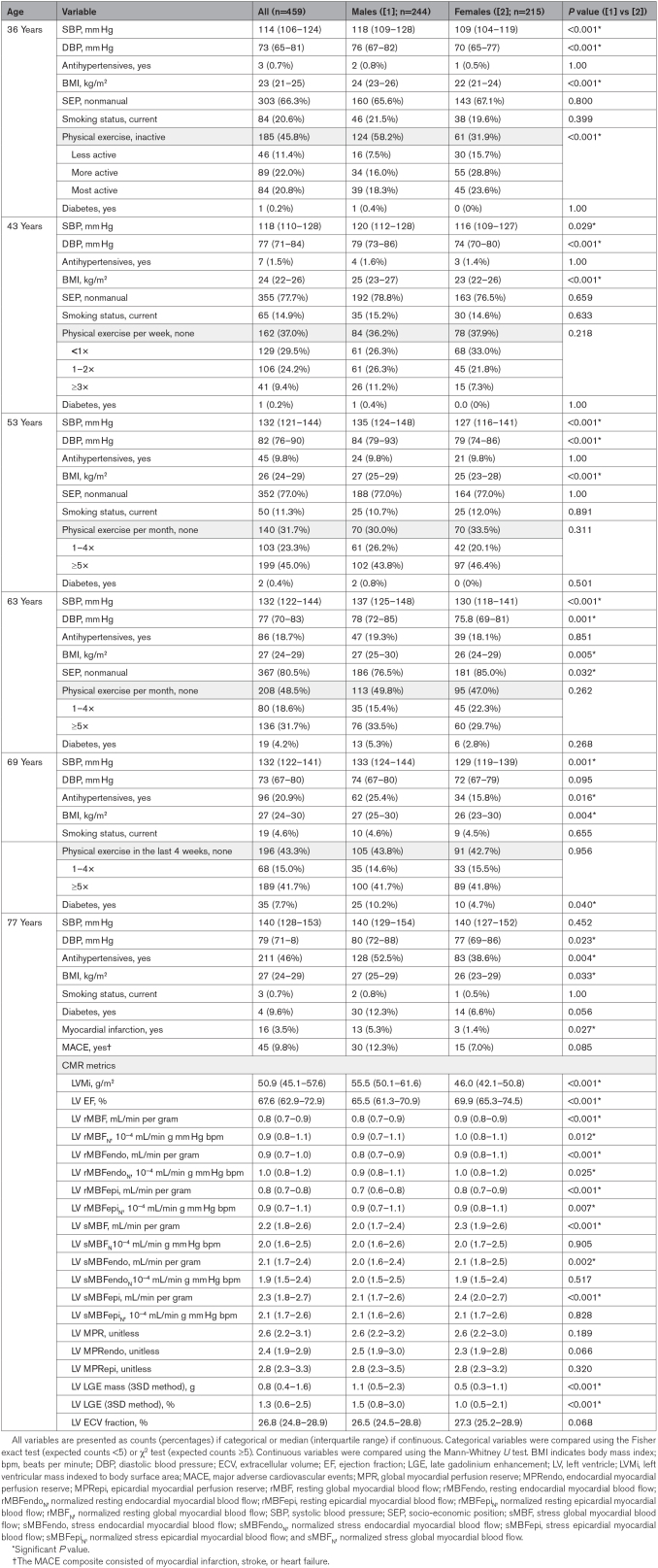
Participant Characteristics Stratified by Sex

### Statistics

All statistical analyses were performed in R (version 4.5.2). Categorical variables were compared using the Fisher exact test (expected counts <5) or the χ^2^ test (expected counts ≥5). Continuous variables were compared using the Mann-Whitney *U* test. Separate analyses were conducted for SBP, DBP, MAP, and pulse pressure. Our main stress CMR perfusion metrics were sMBF_N_ and MPR. For each estimate derived, a 95% CI is provided.

#### Participant Clusters Based on Life-Course BP Trajectories

Latent class mixed models using natural cubic splines (with 36, 43, 53, 63, 69, and 77 years denoted as knots [36 and 77 years were boundary knots and the rest were internal knots]) were used to group MyoFit46 and NSHD participants into clusters, based on their life-course SBP and DBP trajectories from 36 to 77 years, aiming to identify 2 to 5 clusters with sample sizes of >25 participants.

In MyoFit46, separate analyses were conducted for males and females. We compared the stress CMR perfusion metrics at 77 years, across these life-course BP clusters using the Mann-Whitney *U* test.

#### Associations Between Life-Course BP Measures and Stress CMR Perfusion Metrics at 77 Years

We explored the associations between BPs at 36 to 77 years and CMR perfusion at 77 years using generalized linear models with a gamma distribution and log link. Model 1 was unadjusted. Model 2 was adjusted for sex, age at CMR, and for antihypertensive drug use, socio-economic position, body mass index, smoking, physical activity, and diabetes, all measured at the time when the corresponding BP was recorded (or closest available). For analyses at 69 and 77 years, the socio-economic position at ≈63 years was used, as beyond this age most participants were retired. For the analysis at 77 years, physical activity at age 69 was substituted. Of note, since CMRs were performed within the 74 to 78 years window, adjustment for age was used to improve the precision of regression estimates. To explore whether BPs across the life-course exert a legacy effect on myocardial perfusion in later life, independent of concurrent BP levels, model 3 was further adjusted for BP at 77 years. Using generalized additive models, we tried to identify any nonlinear relationships between BPs at 36 to 77 years and CMR perfusion at 77 years.

#### Associations Between Cumulative Life-Course BP Burden and Steepness of BP Increase and Myocardial Perfusion at 77 Years

To account for within-subject repeated BP measures, we used random coefficients mixed-effects natural cubic spline models to derive the BP trajectory across the life-course in each participant. Knots were assigned at 36, 43, 53, 63, 69, and 77 years, giving rise to 5 splines corresponding to the time periods in-between. For each individual, this model was used to estimate the cumulative life-course BP burden, as the mean life-course BP and the area under the BP trajectory curve (AUC_BP_) over age, calculated as $AUC_{BP}=\int_{36\;years}^{77\;years}BP\left(age\right)\;dage$. The annual rates of BP change between 36 and 43, 43 and 53, 53 and 63, 63 and 69, and 69 and 77 years were calculated as BP-rate_x-y_=$AUC_{BP}=\int_{36\;years}^{77\;years}BP\left(age\right)\;dage$, using the actual recorded BPs. Using generalized linear models with a gamma distribution and log link (models 1, 2, and 3 defined as above), we explored whether these BP metrics (mean life-course BP, AUC_BP_, and annual rates of BP change) associate with worse myocardial perfusion at 77 years. For annual rates of BP change models, we additionally adjusted for the baseline BP (eg, the model using SBP-rate_36–43_ was adjusted for SBP at 36 years). To test whether the steepness of the BP increase associating with lower myocardial perfusion is conditional on baseline or end BPs, we tested for the interactions between the annual rates of BP change and the initial and final BPs for each age interval. When testing for the interactions between the rates and the final BPs, models were no longer adjusted for the baseline BPs. To explore whether the steepness of the BP increase is an independent predictor beyond cumulative life-course BP burden, we fitted a model 4, adjusted for AUC_BP_ beyond model 2.

#### Mediation Analyses to Explore Mechanistic Pathways

Using the Imai, Keele, Tingley, and Yamamoto mediation framework,^19^ we explored to what extent the associations between higher life-course BPs and worse stress CMR perfusion metrics at 77 years may be explained by higher BPs associating with a greater LVM index. Similarly, we investigated to what extent the associations between higher life-course BPs and greater LGE or ECV at 77 years may be explained by higher BPs associating with lower myocardial perfusion at 77 years. Full details are presented in the Supplemental Methods.

#### Associations Between CMR Perfusion Metrics and MACE

To understand the potential clinical impact of the detected magnitude of reduced myocardial perfusion linked to higher life-course BPs, we explored the cross-sectional associations between CMR perfusion metrics and a prevalent MACE composite consisting of MI, stroke, and HF. We fitted logistic regression models using the CMR perfusion metrics as the independent variables and the MACE outcome as the dependent variable. The CMR perfusion metrics were minimum-maximum rescaled to be distributed between 0 and 100, so the odds ratio for MACE per 1% lower myocardial perfusion can be calculated.

#### Associations Between Life-Course BPs and Mortality

In NSHD, we used multivariable Cox regression models to test for the associations between life-course BPs and all-cause mortality (models 1 and 2 as above).

#### Sensitivity Analyses

We repeated the main analyses using the BPs corrected for antihypertensive use (by adding 10 mm Hg to SBP and 5 mm Hg to DBP in individuals taking such medications) and after removing all individuals who were on antihypertensives. We explored whether the findings were replicated for the endocardial and epicardial regions (sMBFendo_N_ and sMBFepi_N_), for sMBF (not normalized), and for rMBF metrics. To mitigate data missingness bias, we generated 50 datasets with complete covariate information using predictive mean matching multiple imputation. To verify the robustness of our results, we fitted model 2 in all 50 datasets and aggregated the results across all of them using Rubin’s rule. Finally, we compared the CMR perfusion metrics between individuals on antihypertensives and those with elevated BP (SBP ≥130 mm Hg or DBP ≥90 mm Hg^20^) who were not on antihypertensives. Within the treated group, we further compared the CMR perfusion metrics between those who achieved adequate BP control (SBP <130 mm Hg or DBP <90 mm Hg^20^) and those who did not.

## Results

**Figure 1.**
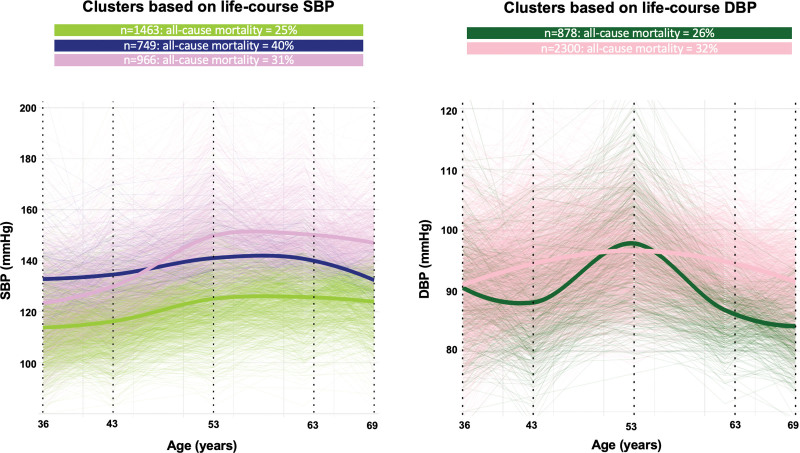
**Life-course trajectory clusters based on systolic (SBPs) and diastolic blood pressures (DBPs) in non-MyoFit46 National Survey of Health and Development (NSHD) study participants.** In NSHD, BPs were recorded at 36, 43, 53, 63, and 69 years (vertical dotted black lines). Latent class mixed models using natural cubic splines (with 43, 53, and 63 years denoted as internal knots) grouped study participants into clusters based on their life-course BP trajectories, with separate analyses conducted for SBP and DBP. Clusters are displayed using bold lines, while individual participant trajectories are also shown using thin lines. All-cause mortality in each cluster is displayed on the top.

Before MyoFit46 recruitment, 1563 (29%) NSHD participants died. Each 10 mm Hg greater SBP at 36 to 53 years could be associated with 6% to 14% higher risk of all-cause mortality (Table S1). NSHD participants with more progressive SBP or DBP trajectories were more likely to die (Figure 1). Of the 505 NSHD participants recruited to MyoFit46, 484 completed their CMR scans. However, both BP at ≥1 time point from 36 to 69 years and stress CMR perfusion at 77 years were available in 459 participants (53% males). The CMR availability flow chart is shown in Figure S2, and the characteristics of the 459 included participants are presented in Table 1. At 36 to 63 years, men had higher SBPs and DBPs than women (all *P*≤0.029). At 69 years, men had a higher SBP (133 versus 129 mm Hg; *P*=0.001), but a similar DBP (74 versus 72 mm Hg; *P*=0.095). Women and men had similar sMBF_N_ (2.0 versus 2.0, *P*=0.905) and MPR (2.6 versus 2.6; *P*=0.189) at 77 years. Only 45 participants experienced our MACE composite. In cross-sectional analyses at 77 years, each 1% lower sMBF_N_ was associated with 3% (95% CI, 1–5; *P*=0.025) higher odds of prevalent MACE, but we found no associations between MPR and MACE (not shown).

### Life-Course BP Trajectories

Individual life-course BP trajectories and overall BP trajectory trends across all participants are shown in Figure S3. Notably, antihypertensive use increased from 45 individuals at 53 years, to 96 at 69 years, and 211 at 77 years, likely explaining some of the observed declines in BPs after 53 years in our clusters.

**Figure 2.**
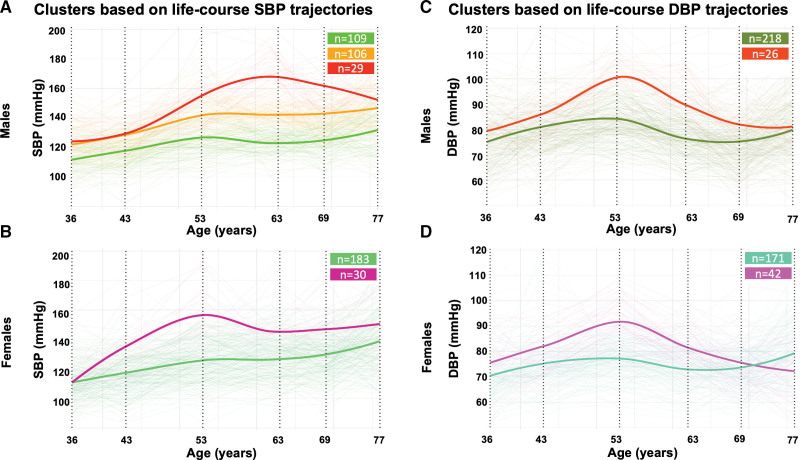
**MyoFit46 clusters based on blood pressure (BP) trajectories across the life-course, stratified by sex.** In MyoFit46, BPs were recorded at 36, 43, 53, 63, 69, and 77 years (vertical dotted black lines). Latent class mixed models using natural cubic splines (with 43, 53, 63, and 69 years denoted as internal knots) grouped study participants into clusters, based on their life-course BP trajectories, with separate analyses conducted for systolic BP (SBP) and diastolic BP (DBP), stratified by sex. Clusters are displayed using bold lines, while individual participant trajectories are also shown using thin lines.

For SBP, we identified 3 male trajectory clusters (Figure 2A): cluster 1 (n=109; normal SBP throughout adulthood), cluster 2 (n=106; more progressive trajectories), and cluster 3 (n=29; steep SBP increases to ≈160 mm Hg from 36 to 63 years). Compared with cluster 1 (sMBF_N_=2.3; MPR=2.8), participants in cluster 2 (sMBF_N_=1.7; MPR=2.6) and cluster 3 (sMBF_N_=1.8; MPR=2.5) had worse myocardial perfusion at 77 years (all *P*≤0.025). For females (Figure 2B), participants with steep SBP increases to ≈160 mm Hg from 36 to 53 years (n=30; sMBF_N_=1.8; MPR=2.0) had worse perfusion compared with those with less progressive trajectories (n=183; sMBF_N_=2.0; MPR=2.6; both *P*≤0.044).

For DBP, males with more progressive trajectories (n=26) had worse sMBF_N_ (1.7 versus 2.0, *P*=0.049) compared with the rest (n=218; Figure 2C). For females (Figure 2D), those with more progressive DBP trajectories (n=42; sMBF_N_=1.9; MPR=2.4) had worse perfusion compared with the other group (n=171; sMBF_N_=2.1; MPR=2.6; both *P*≤0.018).

### Associations Between Life-Course BPs Measures and Stress CMR Perfusion Metrics at 77 Years

**Figure 3.**
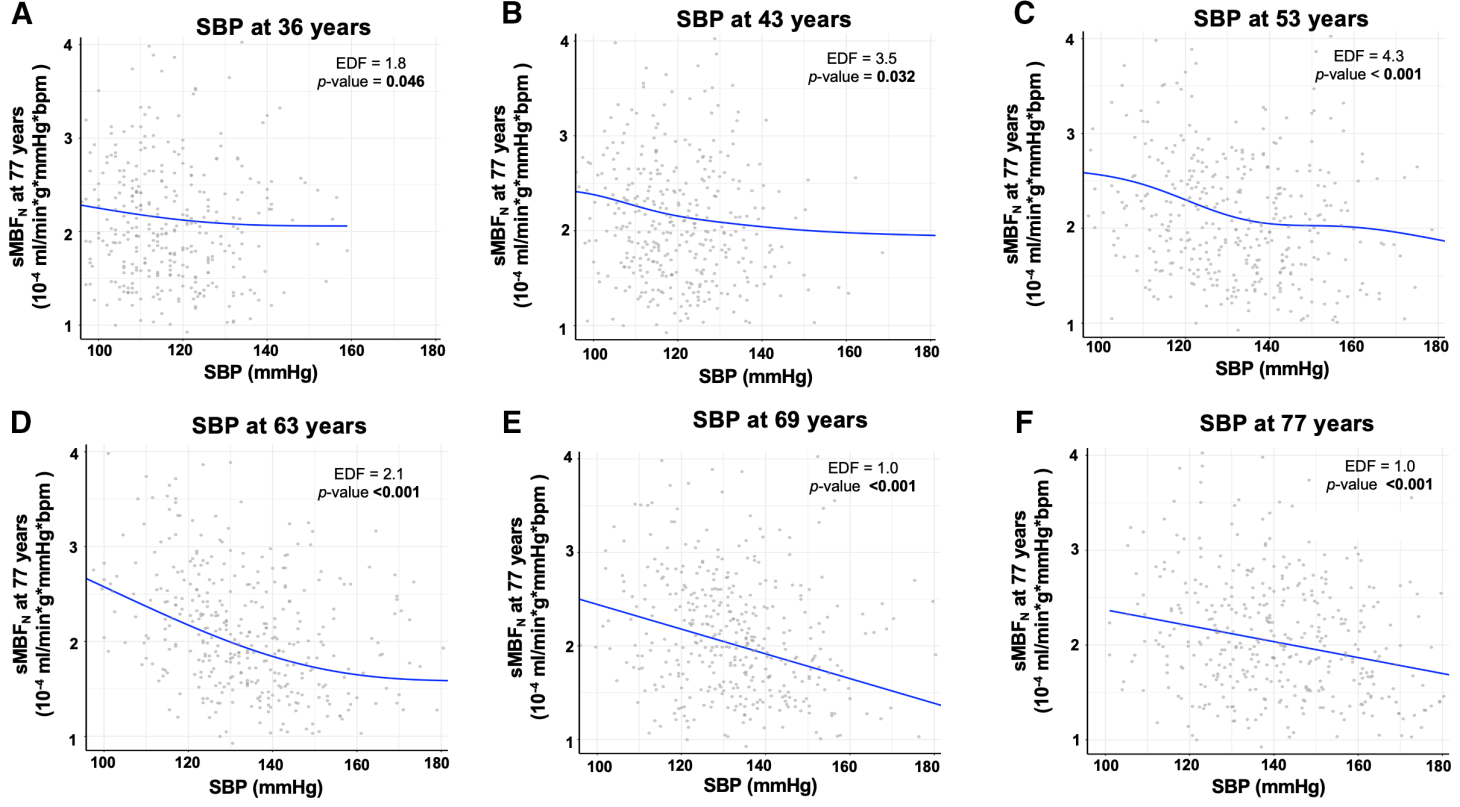
**Relationships between systolic blood pressures (SBPs) across the life-course and normalized stress global myocardial blood flow (sMBF_N_) at 77 years.** Generalized additive models explored any nonlinear relationships between SBPs at 36, 43, 53, 63, 69, and 77 years (expressed in mm Hg) and sMBF_N_ at 77 years (expressed in 10^−4^ mL/min g mm Hg bpm), after adjusting for sex, age at cardiovascular magnetic resonance imaging, and for antihypertensive use, socio-economic position, body mass index, smoking status, physical activity, and diabetes, all measured at the time when the corresponding BP was recorded (or latest available). Significant *P* values are in bold. Effective df (EDF) ≈1 implies a linear relationship, while EDF >1 suggests the presence of nonlinearities.

After adjusting for sex, age at CMR, and for antihypertensive use, socio-economic position, body mass index, smoking status, physical activity, and diabetes, all measured at the time when BP was recorded (or latest available; model 2), each 10 mm Hg higher SBP at 36 to 69 years was associated with 3% to 6% lower sMBF_N_ at 77 years (all *P*≤0.046; Table 2). A 3% to 6% lower sMBF_N_ was associated with 9% to 18% higher odds of prevalent MACE at 77 years. For midlife BPs (43–63 years), associations were curvilinear, with a steeper decrease in sMBF_N_ observed as SBPs rose from 120 to 140 mm Hg, compared to when SBPs rose from 140 to 180 mm Hg (Figure 3). Within the 120 to 140 mm Hg interval, for these midlife BPs, each 10 mm Hg higher SBP was associated with 9% to 12% lower sMBF_N_. Even after adjusting for SBP at 77 years (model 3), SBP measured between 43 and 69 years remained linked to lower sMBF_N_ at 77 years, albeit effect sizes were attenuated by 16% to 25%. Results were similar when using sMBF (not normalized), but effect sizes were generally smaller (Table 2). In model 3, each 10 mm Hg higher SBP at 36 to 69 years was associated with 2% to 4% lower sMBF at 77 years. Consistent with the greater susceptibility of the subendocardium to hypertensive microvascular injury, the % decrease in sMBF_N_ was 0.1% to 1.1% lower per each 10 mm Hg higher SBP endocardially than epicardially (Table S2). Associations persisted when SBPs were corrected for antihypertensive use or if those on antihypertensives were excluded (Table S3). Importantly, each 10 mm Hg greater SBP at 53 to 69 years was associated with 2% to 4% lower rMBF_N_ at 77 years (Table S4). Across 50 complete data sets generated using multiple imputation, results were replicated with similar effect sizes (Table S5).

**Table 2.**
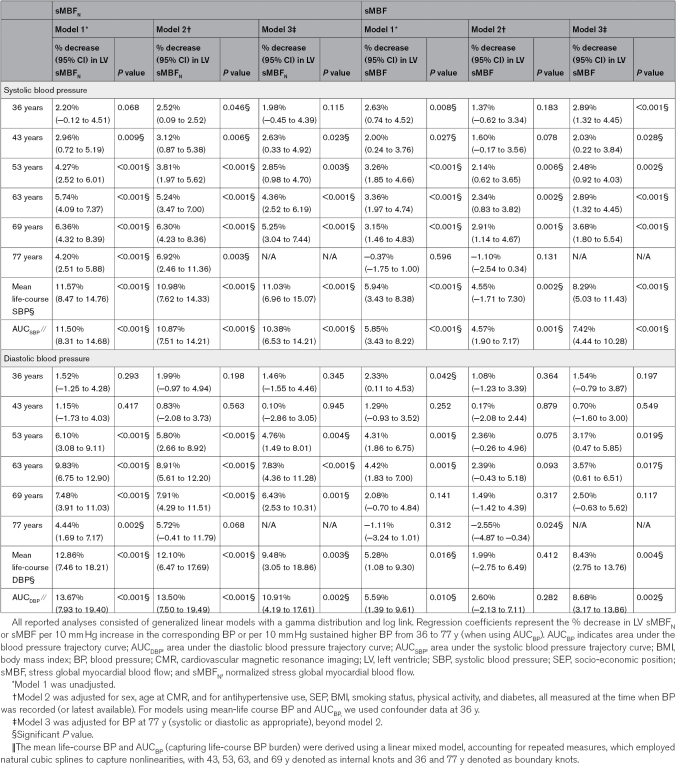
Associations Between Life-Course Systolic and Diastolic Blood Pressures and LV sMBF_N_ or sMBF at 77 Years

In model 3, each 10 mm Hg higher SBP at 43 to 69 years was associated with 3% to 4% lower MPR at 77 years (Table S6). Associations were mostly linear, except at 53 years (Figure S4).

In model 3, each 10 mm Hg higher DBP at 53 to 69 years associated with 5% to 8% lower sMBF_N_ at 77 years (Table 2), while each 10 mm Hg higher DBP at 53 and 63 years associated with 2% to 4% lower MPR at 77 years (Table S6). Associations between life-course MAPs or pulse pressures and CMR perfusion metrics at 77 years are shown in Table S7.

### Cumulative Life-Course BP Burden as a Predictor of Myocardial Perfusion at 77 Years

**Figure 4.**
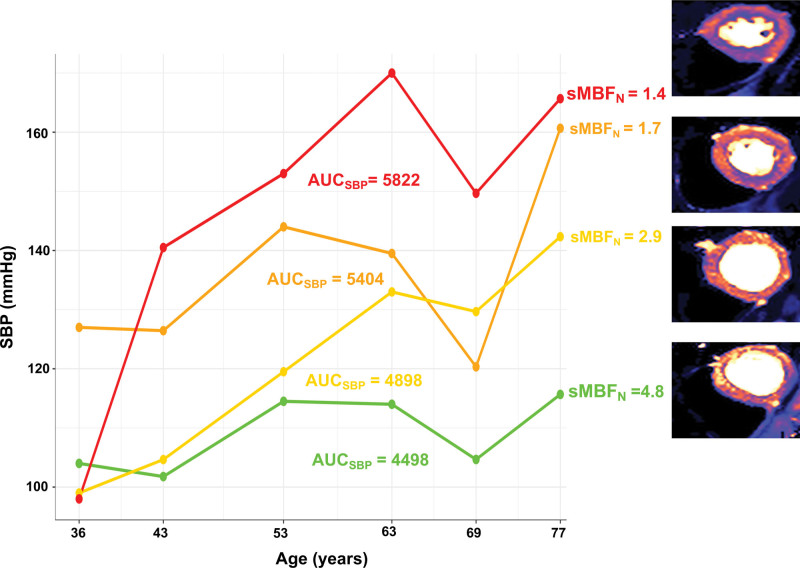
**Exemplar life-course systolic blood pressure (SBP) trajectories and cardiovascular magnetic resonance imaging perfusion maps at 77 years.** In general, individuals with greater areas under the SBP trajectory curve (AUC_SBP_) from 36 to 77 years had lower normalized stress global myocardial blood flows (sMBF_N_) at 77 years.

Having a sustained higher SBP by 10 mm Hg from 36 to 77 years was associated with an 11% (95% CI, 8–14) lower sMBF_N_ (Table 2) and 7% (95% CI, 4–10) lower MPR at 77 years in model 2 (Table S6). These associations persisted after adjusting for SBP at any age from 36 to 77 years. After adjusting for SBP at 77 years, having a sustained higher SBP by 10 mm Hg from 36 to 77 years was associated with 7% (95% CI, 4–10) lower sMBF. Exemplar CMR perfusion maps based on AUC_SBP_ are shown in Figure 4. Results were similar when mean life-course SBP was used instead of AUC_SBP_. In addition, having a sustained higher DBP by 10 mm Hg from 36 to 77 years was associated with an 11% (95% CI, 4–18) lower sMBF_N_ and 9% (95% CI, 3–14) lower sMBF at 77 years, independent of DBP at 77 years and after adjusting for confounders.

### Steepness of BP Increase as a Predictor of Myocardial Perfusion at 77 Years

In model 2, each 1 mm Hg/y steeper SBP increase was linked to lower sMBF_N_ at 77 years by: 2% (95% CI, 1–4) for SBP-rate_36-43_; 4% (95% CI, 2–6) for SBP-rate_43-53_; 6% (95% CI, 4–8) for SBP-rate_53-63_; and 2% (95% CI, 1–3) for SBP-rate_63-69_ (all *P*≤0.018; Table 3). These associations were independent of SBP at 77 years. A 2% to 6% lower sMBF_N_ was associated with 6% to 18% higher odds of prevalent MACE. Importantly, each 1 mm Hg/y steeper SBP-rate_53-63_ and SBP-rate_63-69_ is associated with 2% to 3% lower sMBF_N_, independent of AUC_SBP_. Effect sizes were smaller with fewer significant results when using MPR instead of sMBF_N_ (Table S8). In general, the interactions between these annual rates of SBP change and initial or final BP levels within each age interval were not significant (Table S9).

**Table 3.**
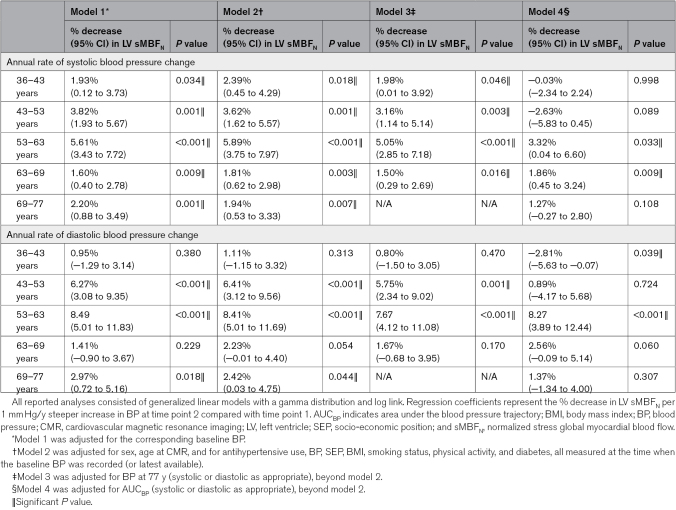
Associations Between Annual Rates of Systolic and Diastolic BP Change and LV sMBF_N_ at 77 Years

### Antihypertensive Use and Myocardial Perfusion at 77 Years

At 36 years, 3 (1%) individuals were on antihypertensives, but 144 (31%) had elevated BPs (SBP ≥130 mm Hg, DBP ≥90 mm Hg; not shown). Those on antihypertensives at 36 years (sMBF_N_=2.6; MPR=3.0) had better myocardial perfusion at 77 years, compared with those with elevated BPs but not on antihypertensives (sMBF_N_=2.0; MPR=2.5), although results were not significant. The 7 (2%) individuals who were on antihypertensives at 43 years (sMBF_N_=2.1; MPR=2.6) had similar myocardial perfusion compared with the 179 (39%) who had elevated BPs but were not on antihypertensives (sMBF_N_=1.9; MPR=2.6), both *P*≥0.788. The 45 (10%) individuals who were on antihypertensives at 53 years (sMBF_N_=2.0; MPR=2.6) had better myocardial perfusion, compared with the 282 (61%) who had elevated BPs but were not on antihypertensives (sMBF_N_=1.8; MPR=2.4), both *P*≤0.048.

Among those on antihypertensives at 53 years, only 16 achieved adequate BP control (SBP <130 mm Hg, DBP <90 mm Hg). Those who achieved control (sMBF_N_=1.9; MPR=2.5) and those who did not (sMBF_N_=1.8; MPR=2.2) had similar myocardial perfusion at 77 years (both *P*≥0.181).

At 63 years, 86 (19%) were on antihypertensives, but only 38 achieved good BP control. Those who had better control (sMBF_N_=2.0; MPR=2.5) had better myocardial perfusion, compared with those who did not (sMBF_N_=1.7; MPR=2.1), both *P*≤0.046.

At 69 years, 96 (21%) were on antihypertensives, but only 65 achieved good BP control, albeit myocardial perfusion metrics were similar (both *P*≥0.485).

### Life-Course BPs and Later-Life LVM, LV Perfusion, and LV Fibrosis

Each 10 mm Hg higher SBPs from 36 to 69 years were associated with 7% to 11% greater LGE mass at 77 years in model 2. Using mediation analyses, we explored to what extent the associations between higher life-course SBPs and more LV LGE at 77 years may be explained by higher SBPs associating with decreased LV perfusion at 77 years. sMBF mediated ≈20% to ≈40% of the associations between life-course SBPs and LGE (Table S10). sMBF was also a significant mediator when DBP was used instead of SBP. We conducted similar analyses for ECV fraction (%), but no mediating effects were found. LVM index was not a significant mediator of the associations between higher life-course BPs and lower myocardial perfusion at 77 years.

## Discussion

The MyoFit46 study of older age community-dwelling British people shows that higher BPs across adulthood, especially between 36 and 69 years, were consistently linked to lower sMBF_N_ by CMR at age 77, even after accounting for BP at 77 years. The steepest declines in sMBF_N_ were observed when midlife SBPs rose from 120 to 140 mm Hg. Individuals with steeper BP increases, or greater cumulative life-course BP burden, had the worst myocardial perfusion at 77 years. These reductions in MBF were clinically important, as lower sMBF_N_ was associated with greater odds of MACE (composite of MI, stroke, and HF) and more LGE on CMR at 77 years.

### Potential Clinical Implications: Early BP Screening and Enforcing Strict Midlife Antihypertensive Adherence

Our findings suggest that BPs from as early as 36 years are linked to lower myocardial perfusion at 77 years. In line with the recommendations of the US Preventive Services Task Force, which advises BP screening for all adults aged ≥18 years, our results emphasize the need to start BP screening early in life. In contrast, the ESC guidelines do not specify a preferred age to initiate screening, potentially delaying hypertension diagnosis.^21^ Importantly, the link between midlife BP and myocardial perfusion at 77 years was independent of BP at 77 years, suggesting that midlife BP may have associations with later-life CMR perfusion, beyond the effects of BP at 77 years. Thus, especially during midlife, a strong adherence to antihypertensives to meet BP targets should be enforced. Indeed, participants who were on antihypertensives had similar or better myocardial perfusion at 77 years, compared with individuals who had elevated BPs (SBP ≥130 mm Hg or DBP ≥90 mm Hg^20^), but were not on antihypertensives. Similarly, among those on antihypertensives, individuals who achieved better BP control (SBP <130 mm Hg or DBP <90 mm Hg^20^) had similar or better myocardial perfusion at 77 years.

### Potential Clinical Implication: Monitoring Life-Course BP Trajectories

Hypertension is often conceptualized as a condition to be managed once it crosses a certain threshold, yet its long-term consequences are shaped by the BP trajectory over time. Cumulative BP, reflected in measures such as the mean life-course BP and the area under the BP trajectory curve, had the strongest association with myocardial perfusion at 77 years. These associations persisted after adjusting for BPs at any age from 36 to 77 years, suggesting that isolated BP readings have less impact on myocardial perfusion than cumulative life-course BP burden metrics. Beyond absolute BP values at any age (even at 36 years), the rate at which BP increases is also relevant. Interestingly, the impact of the steepness of the BP increase is not conditional on initial or final BP levels. These findings suggest that BP management should extend beyond the current static threshold-based system to a more comprehensive approach that considers personalized long-term BP trajectories and cumulative life-course BP burden. Instead of initiating treatment only when cross-sectional BP readings exceed certain thresholds, clinicians may need to monitor BP trajectories serially over time. This would allow physicians to identify steep increases, even if the absolute BP values remain below the current treatment thresholds.This approach could lead to earlier interventions, such as lifestyle modifications or targeted pharmacological therapy, to prevent the long-term cardiovascular consequences associated with elevated BP, such as reduced MBF or MPR. Thus, incorporating cumulative measures into risk assessment may improve cardiovascular prevention by identifying individuals at the highest risk. This approach has shown promise in newly diagnosed cardiovascular artery disease, where better-controlled BP associated with fewer MIs and revascularizations.^22^

We found that the associations between SBPs at 43 to 63 years and myocardial blood flow at 77 years were curvilinear, with steeper decreases as SBPs rose from 120 to 140 mm Hg. Our findings support recent shifts in both ESC and American College of Cardiology/AHA guidelines,^4^ which now recommend initiating antihypertensive treatment at 130 mm Hg in high-risk patients. However, lowering the SBP treatment threshold and target to 120 mm Hg could offer additional benefits in terms of myocardial perfusion in older age.

### Strengths and Limitations

A strength of this study is the age homogeneity of participants, meaning that everybody had similar access to diagnostic and treatment facilities at each age, so participants are more comparable in contrast to a nonaged matched cohort. Our MACE outcome included events accrued up to the time of the CMR, making our analyses between CMR perfusion metrics and prevalent MACE a cross-sectional analysis, rather than a time-to-event analysis. The low number of MIs (n=16) and the lack of gold-standard coronary angiographic data to distinguish MI with nonobstructive coronary arteries from type 1 MI, limited our ability to determine whether the impact of life-course BPs on quantitative CMR perfusion at 77 years is related to microvascular dysfunction or to obstructive cardiovascular artery disease. Although we identified different BP trajectory clusters in males and females, some of these had small sample sizes (n<50), increasing the risk of unstable parameter estimates that may result in clusters which do not adequately capture the true heterogeneity of BP trajectories in the general population. These sex-stratified analyses should be considered exploratory and require validation in larger cohorts. BPs were recorded at 43, 53, and 63 years, and since the average age of menopause is ≈50 years, many female participants likely transitioned through menopause between assessments. Another notable limitation is that sMBF mediated the associations between life-course BPs and LGE, but not with mean global ECV fraction (%). A possible explanation is selection bias, as the timing of the CMR at 77 years excludes participants who may have had the highest life-course BPs, but died before having the chance to be enrolled in MyoFit46. We used the mean global ECV fraction (%), but segment-specific ECV fraction (%), maximum regional ECV fraction (%), synthetic ECV fraction (%), or 95–5 ECV (%) dispersion may better capture interstitial fibrosis in older age. Since this is an epidemiological study, we cannot infer causality. However, our study is the first one to link life-course BP trajectories from 36 to 69 years with reduced myocardial perfusion by CMR at 77 years.

### Conclusions

Higher life-course BPs (as early as 36 years), steeper BP increases (regardless of initial or final BPs), and more years spent at higher BPs were associated with lower myocardial perfusion at 77 years. In a cross-sectional analysis, each 1% lower sMBF_N_ could be linked to 3% higher odds of a prevalent MACE composite consisting of MI, stroke, or HF, suggesting that the associations between life-course BPs and sMBF_N_ may be clinically significant. Our findings emphasize the importance of early life BP screening, integrating life-course BP trajectories for a more personalized cardiovascular risk stratification, rigorous midlife BP control, and considering lower BP treatment thresholds and targets. Moving forward, large prospective clinical trials are needed to evaluate whether treating hypertension based on the steepness of the BP increase or the cumulative life-course BP burden is a superior approach compared to the current threshold-based system.

## Article Information

### Acknowledgments

We are grateful to the National Survey of Health and Development (NSHD) participants who contributed to research over the last eight decades. We would like to thank the wider NSHD team for their data collection efforts and the staff and radiographers at Chenies Mews Imaging Center, who provided help in setting up the cardiovascular magnetic resonance imaging protocol.

### Author Contributions

All authors contributed significantly to the design, implementation, analysis, interpretation, and article writing. However, the study was led by Dr Topriceanu as the first author and Dr Captur as the senior author. The corresponding author attests that all listed authors meet the authorship criteria and that no others meeting the criteria have been omitted.

### Sources of Funding

Sources of funding used for staff salaries, CMR scan costs, consumables, and relevant travel costs required for data collection, analysis and interpretation: British Heart Foundation special project grant (to Dr Captur, SP/20/2/34841). NSHD is supported by the Medical Research Council (Core Unit Level Funding: MC UU 00019/1). Dr Captur is supported by the British Heart Foundation (BHF, MyoFit46 Special program Grant SP/20/2/34841), the BHF Accelerator Award (AA/18/6/34223), the NIHR Invention for Innovation FAST grant scheme (iFAST NIHR205960) and the NIHR UCL Hospitals Biomedical Research Center. Dr Moon is directly and indirectly supported by the UCL Hospitals NIHR BRC at Barts Hospital, respectively. Dr Hughes receives support from the British Heart Foundation, the Economic and Social Research Council, the Horizon 2020 Framework program of the European Union, the National Institute on Aging, the National Institute for Health Research University College London Hospitals Biomedical Research center, the UK Medical Research Council and works in a unit that receives support from the UK Medical Research Council. Swapnanil De was supported by the Wolfson Foundation and the Royal College of Physicians.

### Disclosures

The views expressed in this article are those of the authors, who declare that they have no conflict of interest related to this work. Dr Moon is the chief executive officer of MyCardium AI and has served on advisory boards for Genzyme and Sanofi. Dr Chaturvedi receives funds from AstraZeneca to serve on clinical trial data safety and monitoring committees. The other authors report no conflicts.

### Supplemental Material

Supplemental Methods

Tables S1–S10

Figures S1–S4

## Supplementary Material
