# Supplementary material for: Higher Life-Course Blood Pressure Associates With Reduced Myocardial Perfusion in Older Age: Insights From MyoFit46
Source: Circ Cardiovasc Imaging. 2025 Dec 17;19(2):e019105. doi: 10.1161/CIRCIMAGING.125.019105 (PMC12908645; doi:10.1161/CIRCIMAGING.125.019105)

SUPPLEMENTAL MATERIAL FOR:

Higher life-course blood pressure associates with reduced myocardial perfusion in older age: insights from MyoFit46

Short title: Life-course BP and myocardial perfusion in older age

Constantin-Cristian Topriceanu[1,2,3,4]; Matthew Webber[1,2]; Hunain Shiwani[2,5]; Fiona Chan[1,2]; Emma Martin[1,2]; Debbie Falconer[1,2]; Matthew A Stanley[1,2]; Jonathan Bennett[1,2]; Pablo Gonzalez-Martin[2,6]; Haytham Shah[1,2], Swapnanil De[1,2], Andrew Wong[1,2], Iain Pierce[5], Rhodri H Davies[1,2], Pier D Lambiase[2,5], Nishi Chaturvedi[1,2]; Peter Kellman[7]; Rebecca Hardy[8]; James C Moon[2,5]; Alun D Hughes[1,2]; Gabriella Captur[1,2,3]

1. Unit for Lifelong Health and Ageing at UCL, University College London, London, UK
2. UCL Institute of Cardiovascular Science, University College London, London, UK
3. Cardiology Department, Royal Free Hospital, London, UK
4. Mayo Clinic, Rochester, MN, USA
5. Cardiac MRI Unit, Barts Heart Centre, West Smithfield, London, UK
6. ELEM Biotech SL, Barcelona, Spain
7. National Heart, Lung, and Blood Institute, National Institute of Health, Bethesda, MD, USA
8. School of Sport, Exercise and Health Sciences, Loughborough University, Loughborough, UK

Address for Correspondence:

Gabriella Captur

Consultant Cardiologist in Inherited Heart Muscle Conditions, Senior Clinical Lecturer

Institute of Cardiovascular Science, University College London, London WC1E 6BT, UK

E-mail: gabriella.captur@ucl.ac.uk

Phone No: +442074600595

SUPPLEMENTAL METHODS

Cardiovascular magnetic resonance imaging protocol

All participants had point-of-care creatinine and hematocrit tests immediately prior to the cardiovascular magnetic resonance (CMR) performed at the University College London Bloomsbury Centre for Clinical Phenotyping using a single 3-Tesla [200 mT/m/s x 80 mT/m] MR system (Magnetom Prisma, Siemens) operating VE11C-SP01, with an 18-channel phased-array chest coil and spine array equipped with Gadgetron. Standard long- and short-axis (LAX, SAX) cine imaging were acquired, using breath-held, retro-gated balanced steady-state free precession (bSSFP) sequences, with the following parameters: flip angle 50°; temporal resolution 29.1ms; echo time/spacing 1.25/2.9ms; slice thickness/gap 8.0/2.0mm; field of view 380x285mm; matrix size 256x140; reconstructed voxel size 1.5x1.5x8.0; and cardiac phases 30. Basal, mid and apical left ventricular (LV) co-registered pre- and post-gadolinium-based contrast agent (GBCA) SAX T_1 maps were generated using free-breathing modified Look-locker inversion recovery.

Intravenous adenosine was infused at 140 µg/kg/min for 3-4 minutes prior to the first bolus injection of GBCA, assessing for an adequate response if heart rate (HR) increased by ≥10 beats per minute (bpm) and/or if symptoms developed. In case of suboptimal response (no symptoms and HR did not increase by 10bpm), a higher dose of 175 µg/kg/min was infused for 2 additional minutes. The first dose of gadoterate meglumine (Dotarem, Guerbet, France; 0.05 mmol/kg), a GBCA, was injected via the second canula, at a rate of 4ml/s with 20ml saline. After stopping the adenosine infusion, stress perfusion images were acquired using a free-breathing motion-corrected saturation-recovery bSSFP dual sequence[1], covering 3 LV SAX slices across each heartbeat. Rest perfusion images were acquired without adenosine using the second dose of Dotarem, ~10 minutes after the peak stress was achieved. Peripheral brachial and central aortic blood pressure (BP) measurements were performed, on the right arm, directly in the scanner bore using a CMR-safe cuff with long tube, at baseline, peak adenosine stress and recovery using an oscillometric BP machine (Cardioscope II BP +, USCOM, Australia). Central BP measurements recorded at the time of the stress and rest perfusion acquisitions were used to permit rate pressure product (RPP) normalization ($_N$) of values (RPP = heart rate x central systolic BP). This normalization was performed because not everybody reaches maximal vasodilation at stress since individuals may respond differently to adenosine, which may be especially true for our cohort, consisting of individuals aged 77 years. RPP_N values were scaled using the 10000 constant. Late gadolinium enhancement (LGE) imaging utilized a phase-sensitive inversion recovery technique 10 minutes after the second GBCA dose. Post-GBCA T_1 maps were acquired 15 minutes after GBCA administration at the same slice location, field-of-view and shim settings as the pre-GBCA images, thus generating post-GBCA T_1 maps and extracellular volume (ECV) maps.

CMR perfusion quality assurance

A detailed quality assurance report is being published separately. Briefly, participants were excluded from the 'stress' group if they met at least two of the following criteria to suggest inadequate stress response: absent splenic switch off; insufficient heart rate increment; or insufficient symptoms.

Tissue characterization

Manual epicardial and endocardial contours applied to the basal, mid and apical LV SAX slices were automatically eroded by 20% to avoid confounding by blood-myocardial interface on T_1 and ECV maps. For blood T_1 analysis, a region of interest was drawn in the central LV blood pool for each of the three SAX slices, avoiding papillary muscles. The contours and ROIs were copied from the native T_1 slices onto the corresponding post-GBCA T_1 images. Lastly, manual ECV fraction was calculated as ECV = [Δ(1/ T_1 myocardial)/ Δ(1/ T_1 blood)*((1-hematocrit)].

Statistics

Mediation analyses to explore mechanistic pathways: To calculate the effect of the independent variable onto the mediator (the mediator model), we regressed the BP on the mediator. To calculate the effect of the mediator on the dependent variable (the outcome model), we regressed the mediator on the outcome. To calculate the total effects, we regressed BP on the outcome. This allowed us to derive the total effects, average causal mediation effects (ACME), and average direct effects. The proportion of mediation was calculated as the ratio of ACME to total effects, expressed as a percentage. In these mediation analyses, CMR perfusion metrics were not normalized, to avoid forcing BP twice during effect decomposition.

Table S1. Associations between life-course systolic and diastolic BPs and all-cause mortality in the whole NSHD cohort.

	**Model 1***		**Model 2†**	
	**HR (95% CI)**	***p*-value**	**HR (95% CI)**	***p*-value**
		**Systolic blood pressure**		
**36 years**	1.19 (1.15, 1.24)	**<0.001**	1.14 (1.09, 1.19)	**<0.001**
**43 years**	1.16 (1.12, 1.21)	**<0.001**	1.11 (1.07, 1.16)	**<0.001**
**53 years**	1.12 (1.08, 1.16)	**<0.001**	1.06 (1.02, 1.11)	**0.002**
**63 years**	1.08 (1.02, 1.14)	**0.005**	1.03 (0.97, 1.10)	0.296
**69 years**	0.97 (0.90, 1.05)	0.461	0.94 (0.86, 1.02)	0.125
		**Diastolic blood pressure**		
**36 years**	1.19 (1.13, 1.25)	**<0.001**	1.14 (1.08, 1.20)	**<0.001**
**43 years**	1.15 (1.09, 1.22)	**<0.001**	1.09 (1.03, 1.15)	**0.004**
**53 years**	1.19 (1.12, 1.26)	**<0.001**	1.08 (1.01, 1.16)	**0.026**
**63 years**	1.07 (0.97, 1.19)	0.178	1.03 (0.92, 1.15)	0.652
**69 years**	0.82 (0.73, 0.93)	**0.002**	0.86 (0.75, 0.98)	**0.025**

* Model 1 was unadjusted.

† Model 2 was adjusted for sex, antihypertensive use, SEP, BMI, smoking status, physical activity, and diabetes, all measured at the time when BP was recorded (or latest available).

All reported analyses consisted of Cox regression models. Regression coefficients represent the HR for all-cause mortality risk per 10 mmHg increase in the corresponding BP. Significant *p*-values are in bold.

Abbreviations: *BMI = body mass index; BP = blood pressure; CI = confidence interval*; *HR = hazard ratio; NSHD = National Survey of Heath and Development study; SEP = socio-economic position.*

Table S2. Associations between life-course systolic and diastolic BPs and LV $sMBF_N$ or MPR by CMR at 77 years, with separate analyses for endocardial and epicardial surfaces.

	**$sMBF_N$ at 77 years**				**MPR at 77 years**			
	**$sMBFendo_N$**		**$sMBFepi_N$**		**MPRendo**		**MPRepi**	
	**% decrease (95% CI)**	***p*-value**	**% decrease (95% CI)**	***p*-value**	**% decrease (95% CI)**	***p*-value**	**% decrease (95% CI)**	***p*-value**
**Systolic blood pressure**								
**36 years**	2.73% (0.21, 5.23)	**<0.001**	2.25% (-0.20, 4.67)	0.076	1.32% (-1.10, 3.72)	0.286	0.38% (-2.03, 2.76)	0.761
**43 years**	2.63% (0.32, 4.92)	**0.025**	2.55% (0.31, 4.77)	**0.024**	2.84% (0.82, 4.86)	**0.006**	2.82% (0.83, 4.81)	**0.006**
**53 years**	4.48% (2.57, 6.37)	**<0.001**	3.36% (1.53, 5.17)	**<0.001**	3.63% (1.85, 5.38)	**<0.001**	2.64% (0.86, 4.40)	**0.004**
**63 years**	5.60% (3.76, 7.42)	**<0.001**	4.97% (3.20, 6.73)	**<0.001**	3.75% (1.98, 5.50)	**<0.001**	3.25% (1.45, 5.02)	**<0.001**
**69 years**	6.70% (4.56, 8.84)	**<0.001**	6.05% (3.98, 8.11)	**<0.001**	3.06% (1.00, 5.11)	**0.003**	3.24% (1.17, 5.30)	**0.002**
**77 years**	4.41% (2.53, 6.28)	**<0.001**	3.86% (2.06, 5.65)	**<0.001**	1.02% (-0.79, 2.82)	0.258	0.99% (-0.83, 2.80)	0.273
**Diastolic blood pressure**								
**36 years**	2.34% (-0.72, 5.39)	0.140	1.78% (-1.19, 4.74)	0.245	2.04% (-0.77, 4.83)	0.157	1.32% (-1.46, 4.09)	0.356
**43 years**	0.89% (-2.12, 3.89)	0.551	0.77% (-2.14, 3.68)	0.589	1.74% (-0.83, 4.31)	0.185	1.50% (-1.04, 4.05)	0.249
**53 years**	6.36% (3.06, 9.63)	**<0.001**	5.44% (2.33, 8.52)	**0.001**	3.21% (0.11, 6.28)	**0.043**	2.63% (-0.43, 5.67)	0.095
**63 years**	9.32% (5.88, 12.73)	**<0.001**	8.61% (5.30, 11.90)	**<0.001**	3.29% (-0.04, 6.60)	0.053	3.10% (-0.25, 6.43)	0.068
**69 years**	7.85% (4.07, 11.61)	**<0.001**	8.02% (4.41, 11.60)	**<0.001**	1.10% (-2.27, 4.45)	0.526	2.01% (-1.35, 5.35)	0.246
**77 years**	4.61% (1.54, 7.67)	**0.003**	4.33% (1.42, 7.23)	**0.003**	-1.71% (-4.49, 1.06)	0.224	-1.41% (-4.19, 1.35)	0.315

All reported analyses consisted of generalized linear models with a gamma distribution and log link. Regression coefficients represent the % decrease in $sMBF_N$ or MPR per 10 mmHg increase in the corresponding BP. All analyses were adjusted for sex, age at CMR, and for antihypertensive use, SEP, BMI, smoking status, physical activity, and diabetes, all measured at the time when BP was recorded (or latest available). Significant *p*-values are in bold.
Abbreviations: *BMI = body mass index; BP = blood pressure; CI = confidence interval; CMR = cardiovascular magnetic resonance imaging; LV = left ventricle; MPR = global myocardial perfusion reserve; MPRendo = endocardial myocardial perfusion reserve; MPRepi = epicardial myocardial perfusion reserve; SEP = socio-economic position;* $sMBF_N$ *= normalized stress global myocardial blood flow;* $sMBFendo_N$ *= normalized stress endocardial myocardial blood flow;* $sMBFepi_N$ *= normalized stress epicardial myocardial blood flow.*

Table S3. Associations between life-course systolic and diastolic BPs and LV $sMBF_N$ or MPR by Cat 77 years, after correcting BPs for antihypertensive use or removing participants on antihypertensives.

	**$sMBF_N$ at 77 years**				**MPR at 77 years**			
	**BPs corrected for antihypertensive use***		**Removed participants on antihypertensives†**		**BPs corrected for antihypertensive use***		**Removed participants on antihypertensives†**	
	**% decrease (95% CI)**	***p*-value**	**% decrease (95% CI)**	***p*-value**	**% decrease (95% CI)**	***p*-value**	**% decrease (95% CI)**	***p*-value**
**Systolic blood pressure**								
**36 years**	2.06% (-0.34, 4.43)	0.098	2.48% (0.03, 4.92)	**0.050**	0.75% (-1.57, 3.06)	0.529	0.88% (-1.49, 3.24)	0.470
**43 years**	2.51% (0.31, 4.69)	**0.024**	2.59% (0.35, 4.82)	**0.022**	2.29% (0.33, 4.26)	**0.024**	2.93% (1.01, 4.85)	**0.003**
**53 years**	4.14% (2.43, 5.84)	**<0.001**	4.54% (2.52, 6.54)	**<0.001**	3.12% (1.51, 4.71)	**<0.001**	3.36% (1.56, 5.15)	**<0.001**
**63 years**	5.31% (3.67, 6.94)	**<0.001**	5.51% (3.47, 7.53)	**<0.001**	4.00% (2.36, 5.62)	**<0.001**	3.39% (1.42, 5.34)	**0.001**
**69 years**	6.21% (4.20, 8.20)	**<0.001**	7.82% (5.61, 10.01)	**<0.001**	3.55% (1.57, 5.52)	**<0.001**	4.21% (2.02, 6.39)	**<0.001**
**77 years**	4.27% (2.58, 5.96)	**<0.001**	5.04% (2.76, 7.31)	**<0.001**	1.52% (-0.15, 3.19)	0.069	1.37% (-0.66, 3.38)	0.176
**Diastolic blood pressure**								
**36 years**	1.38% (-1.47, 4.20)	0.353	2.04% (-0.95, 5.02)	0.188	1.49% (-1.16, 4.13)	0.275	1.69% (-1.07, 4.44)	0.234
**43 years**	0.80% (-2.05, 3.64)	0.571	0.92% (-2.01, 3.85)	0.524	1.03% (-1.46, 3.53)	0.423	1.63% (-0.82, 4.09)	0.194
**53 years**	6.47% (3.47, 9.44)	**<0.001**	7.01% (3.58, 10.40)	**<0.001**	3.34% (0.50, 6.16)	**0.022**	3.66% (0.60, 6.70)	**0.021**
**63 years**	9.22% (6.12, 12.30)	**<0.001**	10.34% (6.71, 13.93)	**<0.001**	4.52% (1.41, 7.60)	**0.005**	3.88% (0.34, 7.38)	**0.031**
**69 years**	10.05% (6.34, 13.60)	**<0.001**	10.54% (6.53, 14.52)	**<0.001**	2.27% (-1.00, 5.52)	0.179	2.84% (-0.79, 6.44)	0.128
**77 years**	4.98% (2.16, 7.77)	**<0.001**	7.01% (3.16, 10.83)	**<0.001**	-0.57% (-3.19, 2.04)	0.669	0.46% (-2.79, 3.69)	0.780

* BPs were corrected for antihypertensive use by adding 10 mmHg to the SBP and 5 mmHg to the DBP in those taking such medications.
† Participants taking antihypertensives were removed from the analyses.

All reported analyses consisted of generalized linear models with a gamma distribution and log link. Regression coefficients represent the % decrease in $sMBF_N$ or MPR 10 mmHg increase in the corresponding BP. All analyses were adjusted for sex, age at CMR, and for SEP, BMI, smoking status, physical activity, and diabetes, all measured at the time when BP was recorded (or latest available). Significant *p*-values are in bold.
Abbreviations: *BMI = body mass index; BP = blood pressure; CI = confidence interval; CMR = cardiovascular magnetic resonance imaging; DBP = diastolic blood pressure; LV = left ventricle; MPR = global myocardial perfusion reserve; SEP = socio-economic position;* $sMBF_N$ *= normalized stress global myocardial blood flow; SBP = systolic blood pressure.*

Table S4. Associations between life-course systolic and diastolic BPs and LV $rMBF_N$ at 77 years.

	**$rMBF_N$ at 77 years**	
	**% decrease (95% CI)**	***p*-value**
	**Systolic blood pressure**	
**36 years**	1.86% (-0.28, 3.97)	0.092
**43 years**	0.31% (-1.65, 2.27)	0.755
**53 years**	1.74% (0.03, 3.44)	**0.045**
**63 years**	2.43% (0.79, 4.07)	**0.004**
**69 years**	3.64% (1.76, 5.51)	**<0.001**
**77 years**	3.76% (2.23, 5.29)	**<0.001**
	**Diastolic blood pressure**	
**36 years**	1.43% (-1.16, 4.01)	0.277
**43 years**	1.59% (-0.92, 4.09)	0.208
**53 years**	4.64% (1.82, 7.44)	**0.001**
**63 years**	5.28% (2.25, 8.29)	**0.001**
**69 years**	6.85% (3.68, 10.01)	**<0.001**
**77 years**	7.81% (5.41, 10.20)	**<0.001**

All reported analyses consisted of generalized linear models with a gamma distribution and log link. Regression coefficients represent the % decrease in $rMBF_N$ per 10 mmHg increase in the corresponding BP. All analyses were adjusted for sex, age at CMR, and for antihypertensive use, SEP, BMI, smoking status, physical activity, and diabetes, all measured at the time when BP was recorded (or latest available). Significant *p*-values are in bold.

Abbreviations: *BMI = body mass index; BP = blood pressure; CI = confidence interval; CMR = cardiovascular magnetic resonance imaging; LV = left ventricle;* $rMBF_N$ *= normalized resting global myocardial blood flow; SEP = socio-economic position.*

Table S5. Associations between life-course systolic and diastolic BPs and LV sMBF$_N$ or MPR at 77 years, across 50 datasets with no missing covariates generated with predictive mean matching multiple imputation.

	sMBF$_N$ at 77 years		MPR at 77 years	
	**% decrease (95% CI)**	***p*-value**	**% decrease (95% CI)**	***p*-value**
**Systolic blood pressure**				
**36 years**	2.54% (0.07, 5.0)	**0.044**	0.95% (-1.45, 3.34)	0.436
**43 years**	2.65% (0.44, 4.86)	**0.019**	2.68% (0.70, 4.67)	**0.008**
**53 years**	3.76% (1.90, 5.62)	**<0.001**	2.98% (1.19, 4.76)	**0.001**
**63 years**	5.37% (3.58, 7.16)	**<0.001**	3.52% (1.79, 5.25)	**<0.001**
**69 years**	6.12% (4.05, 8.18)	**<0.001**	3.01% (1.02, 5.00)	**0.003**
**77 years**	3.81% (2.05, 5.58)	**<0.001**	0.76% (-0.98, 2.49)	0.392
**Diastolic blood pressure**				
**36 years**	2.06% (-0.95, 5.05)	0.179	1.75% (-1.04, 4.52)	0.217
**43 years**	0.67% (-2.17, 3.51)	0.641	1.37% (-1.16, 3.89)	0.287
**53 years**	5.69% (2.53, 8.83)	**<0.001**	2.71% (-0.33, 5.75)	0.081
**63 years**	9.17% (5.89, 12.43)	**<0.001**	3.63% (0.44, 6.82)	**0.026**
**69 years**	7.25% (3.69, 10.79)	**<0.001**	1.17% (-2.11, 4.43)	**0.**483
**77 years**	3.86% (1.03, 6.69)	**<0.001**	-2.01% (-4.72, 0.69)	0.144

Our model 2 consisted of a generalized linear model with a gamma distribution and log link, adjusted for sex, age at CMR, and for antihypertensive use, SEP, BMI, smoking status, physical activity, and diabetes, all measured at the time when BP was recorded (or latest available). Among those with stress CMR perfusion, data missingness per covariate was <10%. However, for certain analyses, data missingness in model 2 reached >20%. To mitigate data missingness bias, we generated 50 datasets with complete covariate information using predictive mean matching multiple imputation. To verify the robustness of our results, we fitted model 2 for each analysis in each multiple imputation dataset and aggregated the results across all 50 using Rubin's rule. Regression coefficients represent the % decrease in sMBF$_N$ or MPR per 10 mmHg increase in the corresponding BP.

Abbreviations: *BMI = body mass index; BP = blood pressure; CI = confidence interval; CMR = cardiovascular magnetic resonance imaging; LV = left ventricle; MPR = global myocardial perfusion reserve; SEP = socio-economic position; sMBF$_N$ = normalized stress global myocardial blood flow.*

Table S6. Associations between life-course systolic and diastolic BPs and LV MPR at 77 years.

	Model 1[†]		Model 2[‡]		Model 3[§]	
	**% decrease (95% CI)**	***p*-value**	**% decrease (95% CI)**	***p*-value**	**% decrease (95% CI)**	***p*-value**
**Systolic blood pressure**						
**36 years**	0.44% (-1.82, 2.70)	0.705	0.78% (-1.59, 3.13)	0.523	0.52% (-1.89, 2.90)	0.673
**43 years**	2.43% (0.41, 4.46)	**0.019**	2.83% (0.86, 4.80)	**0.005**	2.78% (0.74, 4.81)	**0.007**
**53 years**	2.70% (1.07, 4.31)	**0.001**	3.02% (1.27, 4.75)	**0.001**	3.05% (1.24, 4.84)	**0.001**
**63 years**	3.27% (1.65, 4.88)	**<0.001**	3.44% (1.68, 5.17)	**<0.001**	3.46% (1.61, 5.29)	**<0.001**
**69 years**	3.03% (1.11, 4.93)	**0.002**	3.12% (1.10, 5.14)	**0.002**	3.08% (0.90, 5.25)	**0.005**
**77 years**	0.73% (-0.85, 2.30)	0.361	0.98% (-0.81, 2.75)	0.270	N/A	N/A
**Mean life-course SBP**	5.67% (2.67, 8.65)	**<0.001**	7.04% (3.65, 10.41)	**<0.001**	8.89% (4.74, 13.00)	**<0.001**
**AUC_{SBP}**	5.88% (2.89, 8.85)	**<0.001**	7.03% (3.74, 10.31)	**<0.001**	8.13% (3.33, 11.91)	**<0.001**
**Diastolic blood pressure**						
**36 years**	1.25% (-1.36, 3.87)	0.349	1.59% (-1.15, 4.32)	0.260	1.32% (-1.45, 4.09)	0.352
**43 years**	0.61% (-1.91, 3.15)	0.635	1.59% (-0.92, 4.10)	0.215	2.03% (-0.51, 4.58)	0.118
**53 years**	2.22% (-0.60, 5.02)	0.125	2.82% (-0.21, 5.82)	0.069	3.74% (0.64, 6.81)	**0.019**
**63 years**	3.87% (0.86, 6.86)	**0.013**	3.15% (-0.14, 6.41)	0.060	4.37% (0.90, 7.81)	**0.013**
**69 years**	1.41% (-1.67, 4.46)	0.374	1.61% (-1.70, 4.89)	0.345	2.32% (-1.24, 5.86)	0.206
**77 years**	1.71 (-0.66, 4.10)	0.169	1.57% (-1.15, 4.30)	0.257	N/A	N/A
**Mean life-course DBP**	2.47% (-2.47, 7.38)	0.334	3.41% (-2.12, 8.90)	0.233	2.34% (-4.06. 8.69)	0.470
**AUC_{DBP}**	3.28% (-1.80, 8.35)	0.214	4.29% (-1.41, 9.99)	0.146	3.44% (-3.04, 9.91)	0.295

[*]The mean life-course BP and AUC_{BP} (capturing life-course BP burden) were derived using a linear mixed model accounting for repeated measures, which employed natural cubic splines to capture non-linearities, with 43, 53, 63, and 69 years denoted as internal knots.

[†]Model 1 was unadjusted.

[‡] Model 2 was adjusted for sex, age at CMR, and for antihypertensive use, SEP, BMI, smoking status, physical activity, and diabetes, all measured at the time when BP was recorded (or latest available). For models using mean-life course BP and AUC_{BP}, we used confounder data at 36 years.

[§] Model 3 was adjusted for BP at 77 years (systolic or diastolic as appropriate), beyond model 2.

All reported analyses consisted of generalized linear models with a gamma distribution and log link. Regression coefficients represent the % decrease in MPR per 10mmHg increase in the corresponding BP or per 10mmHg sustained higher BP from 36 to 77 years (when using AUC_{BP}). Significant *p*-values are in bold.

Abbreviations: AUCBP = *area under the blood pressure trajectory curve;* AUCDBP = *area under the diastolic blood pressure trajectory curve;* AUCSBP = *area under the systolic blood pressure trajectory curve; BMI = body mass index; BP = blood pressure; CI = confidence interval; CMR = cardiovascular magnetic resonance imaging; DBP = diastolic blood pressure; LV = left ventricle; MPR = global myocardial perfusion reserve; N/A = not applicable; SBP = systolic blood pressure; SEP = socio-economic position.*

Table S7. Associations between life-course mean arterial pressure and pulse pressure and LV $sMBF_N$ or MPR at 77 years.

	**$sMBF_N$ at 77 years**		**MPR at 77 years**	
	**% decrease (95% CI)**	***p*-value**	**% decrease (95% CI)**	***p*-value**
**Mean arterial pressure**				
**36 years**	2.58% (-0.42, 5.56)	0.096	1.50% (-1.33, 4.32)	0.302
**43 years**	1.88% (-0.94, 4.69)	0.179	2.47% (0.02, 4.93)	**0.049**
**53 years**	5.61% (2.92, 8.27)	**<0.001**	3.56% (0.98, 6.13)	**0.008**
**63 years**	8.09% (5.38, 10.77)	**<0.001**	4.14% (1.43, 6.83)	**0.003**
**69 years**	8.58% (5.49, 11.65)	**<0.001**	3.00% (0.07, 5.92)	**0.046**
**77 years**	5.18% (2.57, 7.77)	**<0.001**	-0.24% (-2.75, 2.25)	0.846
**Pulse pressure**				
**36 years**	1.93% (-1.36, 5.19)	0.255	-0.69% (-3.78, 2.37)	0.665
**43 years**	4.75% (1.42, 8.05)	**0.006**	4.06% (1.14, 6.94)	**0.007**
**53 years**	4.04% (1.26, 6.80)	**0.005**	4.60% (1.98, 7.18)	**0.001**
**63 years**	6.06% (3.31, 8.78)	**<0.001**	3.55% (1.35, 5.74)	**0.002**
**69 years**	7.44% (4.40, 10.44)	**<0.001**	5.21% (2.28, 8.12)	**<0.001**
**77 years**	4.82% (2.22, 7.39)	**<0.001**	3.39% (0.86, 5.91)	**0.007**

All reported analyses consisted of generalized linear models with a gamma distribution and log link. Regression coefficients represent the % decrease in $sMBF_N$ or MPR per 10mmHg increase in the corresponding BP. All analyses were adjusted for sex, age at CMR, and for antihypertensive use, SEP, BMI, smoking status, physical activity, and diabetes, all measured at the time when BP was recorded (or latest available). Significant *p*-values are in bold.

Abbreviations: *BMI = body mass index; BP = blood pressure; CI = confidence interval; CMR = cardiovascular magnetic resonance imaging; LV = left ventricle; MPR = global myocardial perfusion reserve; SEP = socio-economic position;* $sMBF_N$ *= normalized stress global myocardial blood flow.*

Table S8. Associations between annual rates of systolic and diastolic BP change and LV MPR at 77 years.

	Model 1*		Model 2†		Model 3‡		Model 4§	
	**% decrease (95% CI)**	***p*-value**	**% decrease (95% CI)**	***p*-value**	**% decrease (95% CI)**	***p*-value**	**% decrease (95% CI)**	***p*-value**
**Annual rate of systolic blood pressure change**								
**36 to 43 years**	1.79% (0.06, 3.49)	**0.038**	1.61% (-0.27, 3.47)	0.087	1.64% (-0.31, 3.54)	0.091	-0.55% (-2.80, 1.65)	0.622
**43 to 53 years**	2.32% (0.49, 4.11)	**0.015**	2.62% (0.75, 4.44)	**0.008**	2.73% (0.84, 4.57)	**0.006**	1.07% (-1.83, 3.87)	0.465
**53 to 63 years**	2.12% (0.34, 3.85)	**0.019**	2.56% (0.79, 4.29)	**0.004**	2.60% (0.77, 4.39)	**0.005**	2.81% (0.09, 5.45)	**0.047**
**63 to 69 years**	0.57% (-1.07, 2.19)	0.483	0.57% (-1.08, 2.19)	0.488	0.67% (-1.04, 2.33)	0.435	1.27% (-0.65, 3.15)	0.183
**69 to 77 years**	0.09% (-1.18, 1.33)	0.865	-0.04% (-1.46, 1.35)	0.951	N/A	N/A	-0.57% (-2.15, 0.98)	0.462
**Annual rate of diastolic blood pressure change**								
**36 to 43 years**	-0.38% (-2.54, 1.74)	0.719	-0.03% (-2.22, 2.11)	0.978	0.10% (-2.10, 2.25)	0.928	-0.87% (-3.55, 1.74)	0.507
**43 to 53 years**	2.29% (-0.88, 5.34)	0.158	2.99% (-0.19, 6.07)	0.066	3.91% (0.72, 6.98)	**0.017**	3.60% (-0.90, 7.90)	0.119
**53 to 63 years**	2.82% (-0.38, 5.91)	0.088	2.84% (-0.36, 5.92)	0.083	3.78% (0.50, 6.95)	**0.025**	4.23% (0.20, 8.08)	**0.042**
**63 to 69 years**	-0.86% (-3.36, 1.58)	0.450	-0.82% (-3.34, 1.64)	0.525	-0.10 (-2.70, 2.42)	0.938	0.23% (-2.65, 3.03)	0.875
**69 to 77 years**	-1.38% (-3.45, 0.64)	0.191	-1.52% (-3.81, 0.70)	0.184	N/A	N/A	-2.35% (-5.00, 0.23)	0.074

* Model 1 was adjusted for the corresponding baseline BP.

† Model 2 was adjusted for sex, age at CMR, and for antihypertensive use, BP, SEP, BMI, smoking status, physical activity, and diabetes, all measured at the time when the baseline BP was recorded (or latest available).

‡ Model 3 was adjusted for BP at 77 years (systolic or diastolic as appropriate), beyond model 2.

§ Model 4 was adjusted for AUC_{BP} (systolic or diastolic as appropriate), beyond model 2.

All reported analyses consisted of generalized linear models with a gamma distribution and log link. Regression coefficients represent the % decrease in MPR per 1mmHg/year steeper increase in BP at time point 2 compared with time point 1. Significant *p*-values are in bold.

Abbreviations: AUC_{BP} = *area under the blood pressure trajectory; BMI = body mass index; BP = blood pressure; CI = confidence interval; CMR = cardiovascular magnetic resonance imaging; DBP = diastolic blood pressure; LV = left ventricle; MPR = global myocardial perfusion reserve; N/A = not applicable; SBP = systolic blood pressure; SEP = socio-economic position.*

Table S9. Interactions between annual rates of systolic and diastolic BP change and initial or final BP levels, when testing for the associations between these rates and LV $sMBF_N$ or MPR at 77 years.

	**$sMBF_N$ at 77 years**				**MPR at 77 years**			
	**Initial BP**		**Final BP**		**Initial BP**		**Final BP**	
	**Initial BP age**	**Interaction *p*-value**	**Final BP age**	**Interaction *p*-value**	**Initial BP age**	**Interaction *p*-value**	**Final BP age**	**Interaction *p*-value**
**Annual rate of systolic blood pressure change**								
**36 to 43 years**	36 years	0.865	43 years	0.143	36 years	0.791	43 years	**0.040**
**43 to 53 years**	43 years	0.412	53 years	0.340	43 years	0.719	53 years	0.215
**53 to 63 years**	53 years	0.418	63 years	0.487	53 years	0.851	63 years	0.532
**63 to 69 years**	63 years	0.070	69 years	0.265	63 years	0.498	69 years	0.121
**69 to 77 years**	69 years	0.434	77 years	0.762	69 years	0.589	77 years	0.358
**Annual rate of diastolic blood pressure change**								
**36 to 43 years**	36 years	0.505	43 years	**0.018**	36 years	0.699	43 years	**0.043**
**43 to 53 years**	43 years	0.098	53 years	0.549	43 years	0.947	53 years	0.357
**53 to 63 years**	53 years	0.569	63 years	0.098	53 years	0.532	63 years	0.431
**63 to 69 years**	63 years	0.226	69 years	0.770	63 years	0.813	69 years	0.485
**69 to 77 years**	69 years	0.527	77 years	0.199	69 years	0.785	77 years	0.471

All reported analyses consisted of generalized linear models with a gamma distribution and log link. All analyses were adjusted for sex, age at CMR, and for antihypertensive use, SEP, BMI, smoking status, physical activity, and diabetes, all measured at the time when BP was recorded (or latest available). For the associations between the annual rates of systolic and diastolic BP change and $sMBF_N$ or MPR, we tested the interaction between these rates and the initial or final BP levels for the corresponding age interval. Significant *p*-values are in bold.

Abbreviations*: BMI = body mass index; BP = blood pressure; CMR = cardiovascular magnetic resonance imaging; LV = left ventricle; MPR = global myocardial perfusion reserve; SEP = socio-economic position;* $sMBF_N$ *= normalized stress global myocardial blood flow.*

Table S10. Mediation analyses.

	Systolic BP		Diastolic BP	
	**Proportion of mediation (95% CI)**	***p*-value**	**Proportion of mediation (95% CI)**	***p*-value**
**Hypothesis: ↑ BP → ↑ LVMi → ↓ LV sMBF**				
**36 years**	8% (-5, 32)	0.204	6% (-18, 43)	0.272
**43 years**	6% (-2, 41)	0.200	18 (-115, 142)	0.236
**53 years**	8% (-6, 27)	0.260	14% (-5, 50)	0.184
**63 years**	8% (-7, 26)	0.310	11% (1, 34)	**0.048**
**69 years**	12% (-6, 38)	0.148	11% (-63, 110)	0.310
**77 years**	-88% (-686, 686)	0.556	-22% (-261, 138)	0.292
**Mean life-course BP**	7% (-10, 31)	0.440	18% (-3, 132)	0.100
**AUC$_{BP}$**	7% (-10, 29)	0.480	16% (-26, 81)	0.104
**Hypothesis: ↑ BP → ↓ LV sMBF → ↑ LV LGE (%)**				
**36 years**	21% (5, 70)	**0.008**	26% (-68, 141)	0.128
**43 years**	27% (1, 116)	**0.046**	40% (-917, 364)	0.530
**53 years**	37% (13, 102)	**0.004**	29% (6, 105)	**0.016**
**63 years**	26% (12, 55)	**<0.001**	27% (10, 126)	**0.016**
**69 years**	44% (-175, 362)	0.080	110% (-388, 609)	0.780
**77 years**	-40% (-235, 326)	0.960	74% (-294, 240)	0.730
**Mean life-course BP**	26% (10, 66)	**<0.001**	24% (6, 126)	**0.036**
**AUC$_{BP}$**	26% (10, 58)	**<0.001**	24% (2, 123)	**0.044**
**Hypothesis: ↑ BP → ↓ LV sMBF → ↑ LV ECV fraction (%)**				
**36 years**	-60% (-447, 394)	0.880	-17% (-185, 179)	0.510
**43 years**	-15% (-222, 176)	0.570	-10% (-97, 93)	0.910
**53 years**	-125% (-904, 403)	0.910	-70% (-308, 334)	0.860
**63 years**	149% (-862, 493)	0.720	38% (-314, 440)	0.860
**69 years**	22% (-155, 193)	0.380	18% (-132, 195)	0.610
**77 years**	-11% (-111, 69)	0.880	24% (-149, 149)	0.980
**Mean life course BP**	337% (-387, 533)	0.880	-43% (-346, 393)	0.840
**AUC$_{BP}$**	936% (-405, 459)	0.910	-48% (-94, 69)	0.810

Using the Imai, Keele, Tingley, and Yamamoto mediation framework, we explored to what extent the associations between life-course BP measures and stress CMR perfusion metrics may be explained by BP associating with a greater LVMi, using mediation analyses. Similarly, we explored to what extent the associations between these life-course BPs and LV LGE and ECV may be explained by BP associating with decreased myocardial perfusion. To calculate the total effects, we regressed the exposure on the outcome. To calculate the effect of the independent variable onto

the mediator (the mediator model), we regressed the exposure on the mediator. To calculate the effect of the mediator on the dependent variable (the outcome model), we regressed the mediator on the outcome outcomes. All analyses were adjusted for sex, age at CMR, and for antihypertensive use, SEP, BMI, smoking status, physical activity, and diabetes, all measured at the time when BP was recorded (or latest available). For models using mean-life course BP and AUC_{BP}, we used confounder data at 36 years. For each estimate, 95% CIs were calculated using nonparametric bootstrapping. Significant *p*-values are in bold.
Abbreviations: *AUC_{BP} = area under the blood pressure trajectory curve; BMI = body mass index; BP = blood pressure; CI = confidence interval; CMR = cardiovascular magnetic resonance imaging; ECV = extracellular volume; LGE = late gadolinium enhancement; LV = left ventricular; LVMi = left ventricular mass indexed to body surface area; SEP = socio-economic position; sMBF = stress global myocardial blood flow.*

Figure S1. NSHD participants lost before MyoFit46 recruitment.
Abbreviations NSHD = National Survey of Health and Development study.

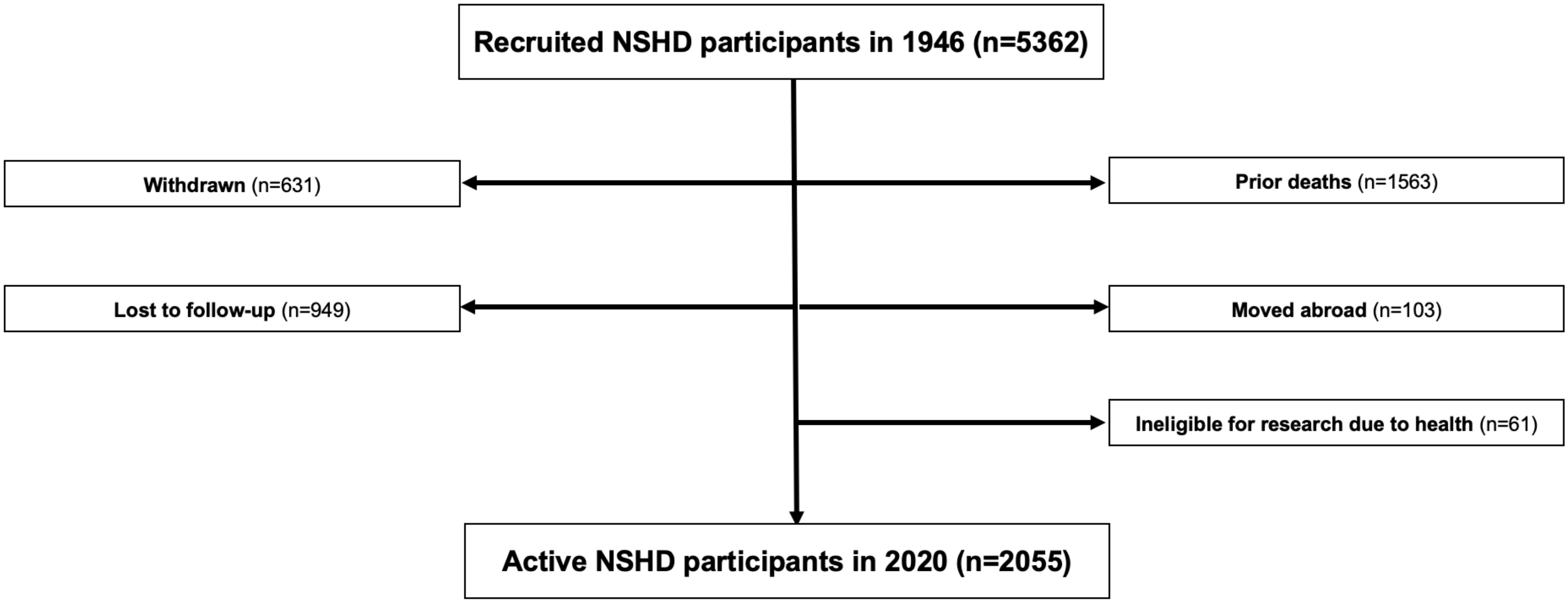

Figure S2. CMR perfusion availability flow chart.

Abbreviations: *AF = atrial fibrillation; BMI = body mass index; CMR = cardiovascular magnetic resonance imaging; ECG = electrocardiogram; GBCA = gadolinium-based contract agents.*

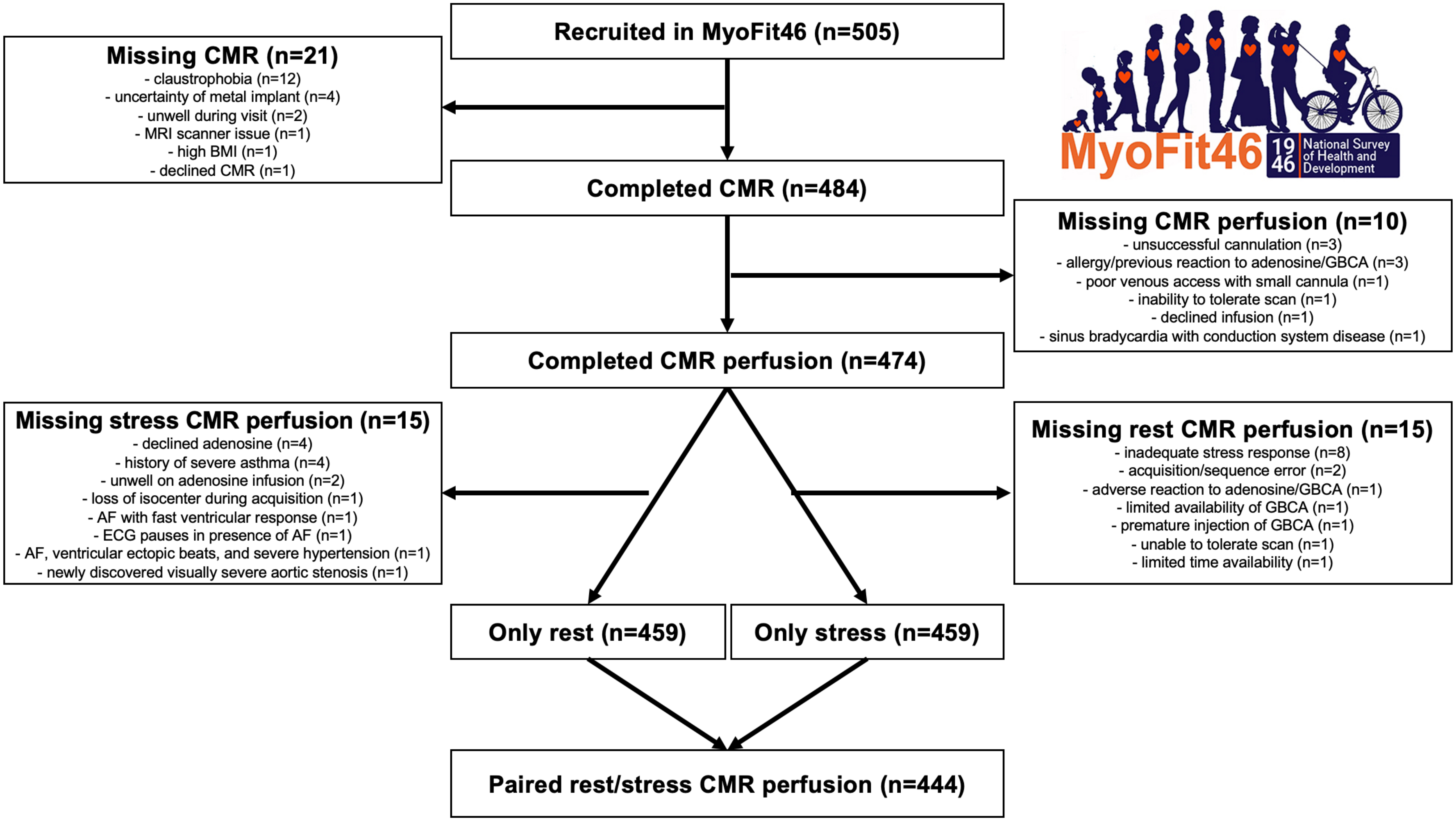

Figure S3. Life-course BP trajectories in MyoFit46.

In MyoFit46, BPs were recorded at 36, 43, 53, 63, 69, and 77 years (vertical dotted black lines). A linear mixed model employing natural cubic splines to capture non-linearities (43, 53, 63, and 69 years were denoted as knots) was used to model the life-course of SBP and DBP trajectories, accounting for repeated measures. The overall trends across MyoFit46 are shown in red. Individual participant trajectories are also shown using thin blue lines.

Abbreviations: *BP = blood pressure; DBP = diastolic blood pressure; SBP = systolic blood pressure.*

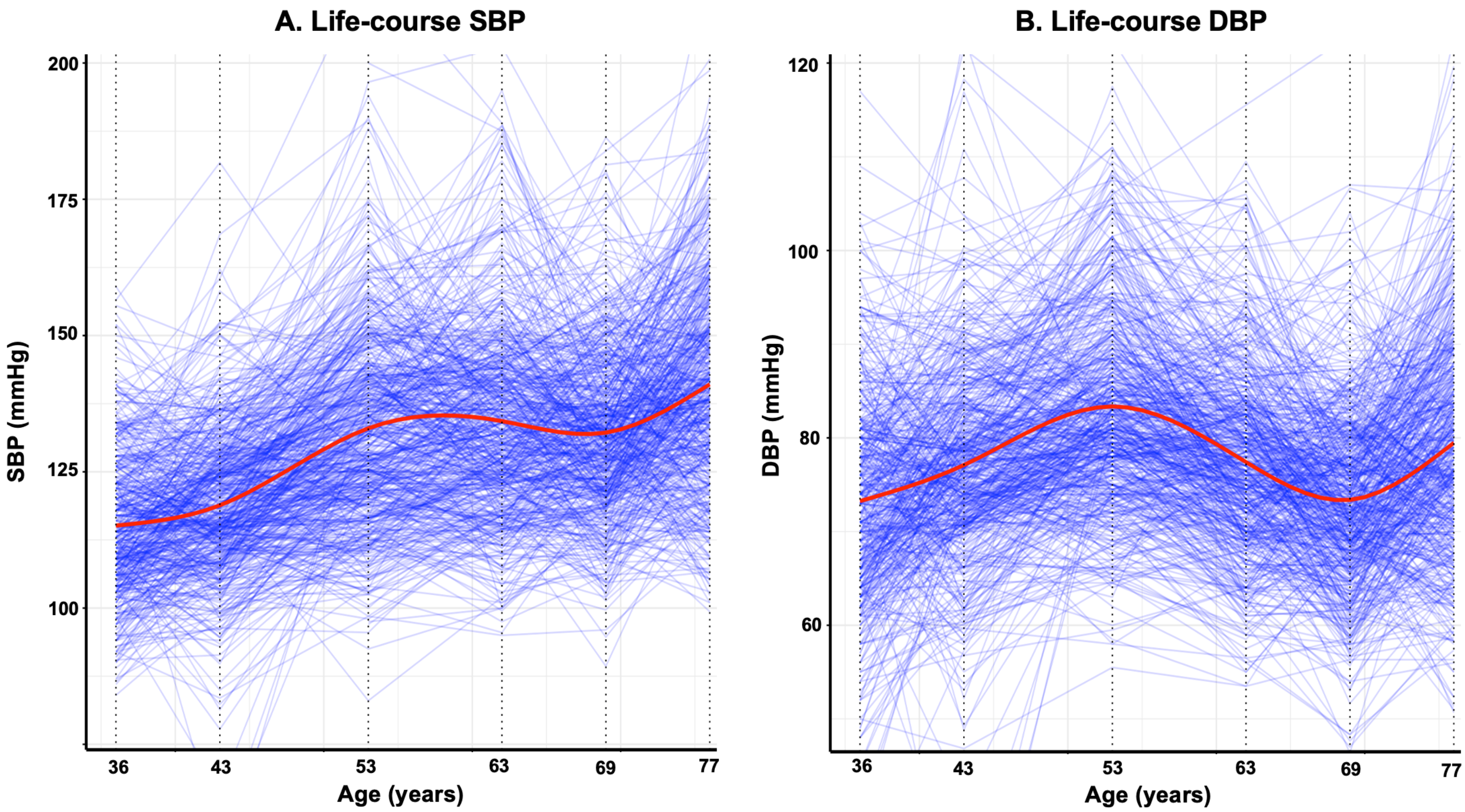

Figure S4. Relationships between life-course SBPs and LV MPR at 77 years.

Generalized additive models explored any non-linear relationships between SBPs at 36, 43, 53, 63, 69, and 77 years (expressed in mmHg) and MPR at 77 years (unitless), after adjusting for sex, age at CMR, and for antihypertensive use, SEP, BMI, smoking status, physical activity, and diabetes, all measured at the time when BP was recorded (or latest available). Significant *p*-values in bold. EDF ~1 implies a linear relationship, while EDF >1 suggests the presence of non-linearities.

Abbreviations*: BMI = body mass index; BP = blood pressure; bpm = beats per minute; CMR = cardiovascular magnetic resonance imaging; EDF = effective degrees of freedom; LV = left ventricle; MPR =global myocardial perfusion reserve; SBP = systolic blood pressure; SEP = socio-economic position.*

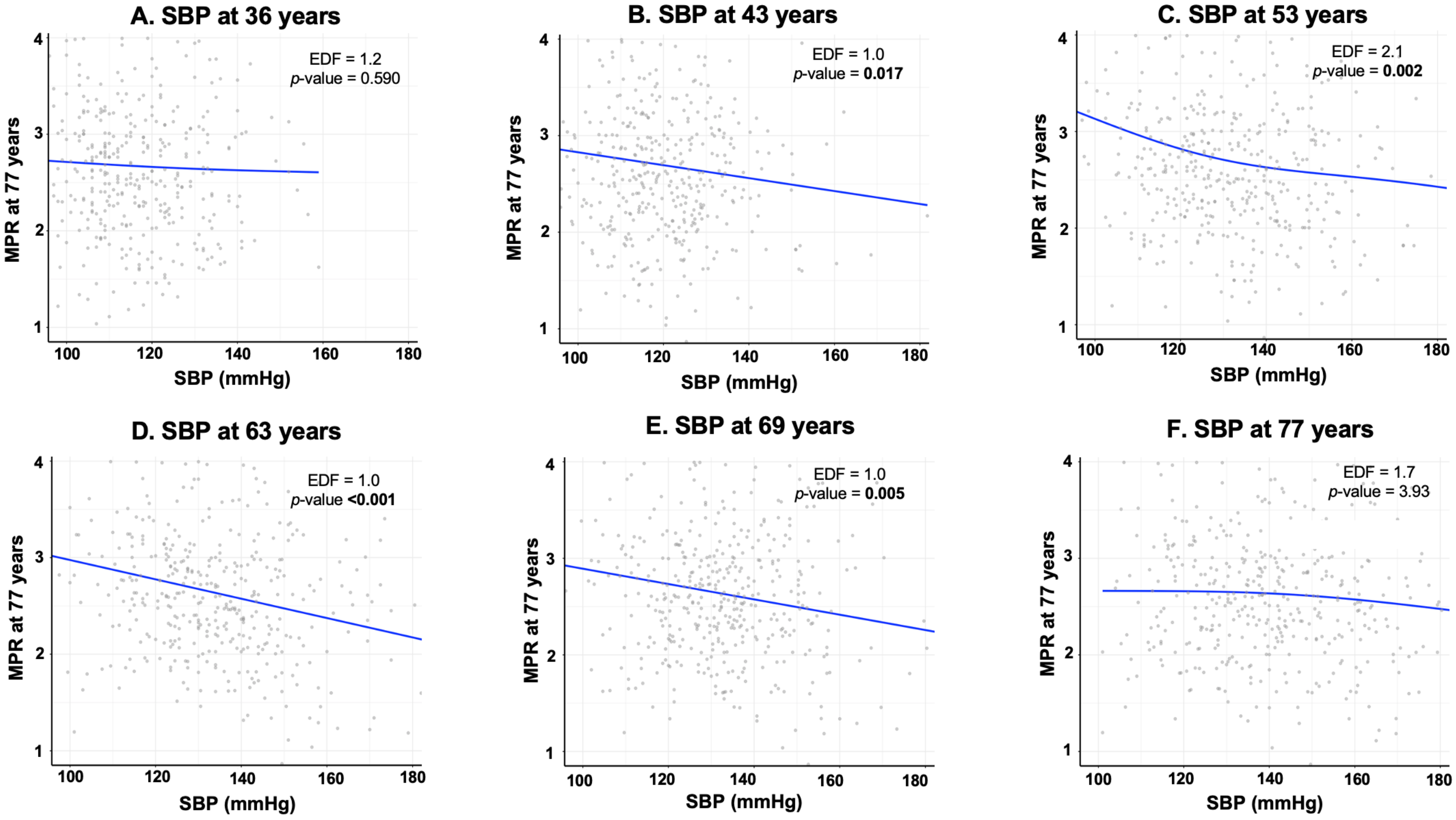

Supplement: Supplementary file 1 [file hci-19-e019105-s001.pdf]
